# Advances of polyolefins from fiber to nanofiber: fabrication and recent applications

**DOI:** 10.1186/s11671-023-03945-y

**Published:** 2024-02-06

**Authors:** Mohammad Zakaria, M. A. Rahman Bhuiyan, Md. Shakawat Hossain, N. M.-Mofiz Uddin Khan, Md. Abdus Salam, Koji Nakane

**Affiliations:** 1 Department of Textile Engineering, Dhaka University of Engineering and Technology, Gazipur, 1707 Bangladesh; 2 Frontier Fiber Technology and Science, University of Fukui, Fukui, 910-8507 Japan; 3 Department of Textile Engineering, Khulna University of Engineering and Technology, Khulna, Bangladesh; 4 Department of Chemistry, Dhaka University of Engineering and Technology, Gazipur, 1707 Bangladesh; 5 Department of Research and Development, Epyllion Fabrics Ltd., Epyllion Group, Gazipur, 1703 Bangladesh

**Keywords:** Polyolefins, High performance fiber, Electrospinning, Filtration and separation, Biomedical engineering, Protective clothing

## Abstract

Polyolefins are a widely accepted commodity polymer made from olefinic monomer consisting of carbon and hydrogen. This thermoplastic polymeric material is formed through reactive double bonds of olefins by the addition polymerization technique and it possesses a diverse range of unique features for a large variety of applications. Among the various types, polyethylene and polypropylene are the prominent classes of polyolefins that can be crafted and manipulated into diversified products for numerous applications. Research on polyolefins has boomed tremendously in recent times owing to the abundance of raw materials, low cost, lightweight, high chemical resistance, diverse functionalities, and outstanding physical characteristics. Polyolefins have also evidenced their potentiality as a fiber in micro to nanoscale and emerged as a fascinating material for widespread high-performance use. This review aims to provide an elucidation of the breakthroughs in polyolefins, namely as fibers, filaments, and yarns, and their applications in many domains such as medicine, body armor, and load-bearing industries. Moreover, the development of electrospun polyolefin nanofibers employing cutting-edge techniques and their prospective utilization in filtration, biomedical engineering, protective textiles, and lithium-ion batteries has been illustrated meticulously. Besides, this review delineates the challenges associated with the formation of polyolefin nanofiber using different techniques and critically analyzes overcoming the difficulties in forming functional nanofibers for the innovative field of applications.

**Graphical abstract:**

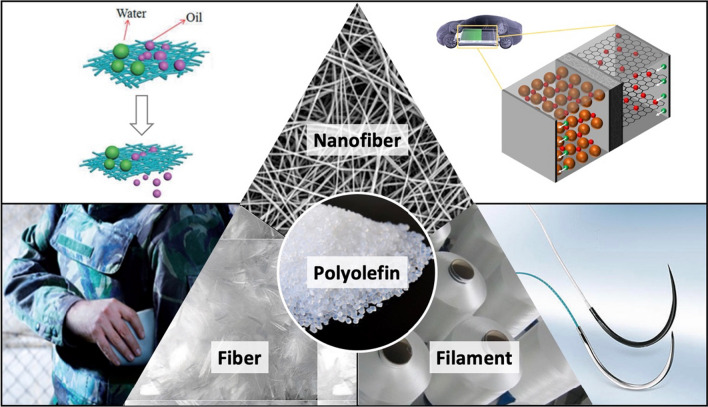

## Introduction

The word “olefins” derive from the French “oléfiant” (oil-forming) and commonly known as “alkenes” (ethylene, propylene, isoprenes, butenes, etc.) are the hydrocarbon with at least one carbon–carbon double bond in their structure [1]. The molecules with a double bond at the first (alpha-) carbon are denoted as alpha (α-) olefins. Polyolefins are thermoplastic polymers formed by the addition polymerization of these reactive double bonds of olefins. The natural carbon supplier crude oil and gas are the main resources of these monomers [2]. Polyolefins are commonly perceived as commodity polymers, have a diverse range of unique features, are inexpensive, and have a large variety of applications [3]. The chemical nature of polyolefins is more advantageous to other plastics, such as polyurethanes, polyamides, and polyvinyl chloride (PVC) for their very simple composition of carbon and hydrogen [4].

Polyolefins derive their excellent physical properties from a large variety of molecular chain arrangements with branching. Branching is caused by the radical transfer that highly influences the physical properties and ensures a variety of olefinic polymer sets with varied expedient functionalities [5]. The recyclability of polyolefins is one of the key advantages, which is accelerating the zero-landfill milestone of modern sustainable earth [6, 7]. Among various types, polyethylene (polymerized ethylene) and polypropylene (PP) (polymerized propylene) are well-known as widely used polyolefins [8]. Worldwide abundant resources of crude oil and natural gas ensure an easy and large volume supply of raw materials for polyolefin production, however, depletion of resources might be an issue in the future [9]. The global demand for polyolefins is increasing day by day due to their low cost, availability with a huge volume, lightweight, large variety of functionalities, high chemical resistance, and promising physical properties. With a clear tendency of growth, around 63% of annual global polymer demand is fulfilled by polyolefins that cover the daily needs of nearly every aspects of our modern lives [10–12]. Only a mere change in fiber diameter to nanometer ranges results in an sharp increase in specific surface area, surface functions, cohesiveness, and even mechanical properties [13–16]. During filtration or separation, the outstanding surface covering provided by the nanofiber's high surface-to-volume ratio serves as a potential barrier against the targeted fine particles and microorganisms [17]. Due to these characteristics, polyolefins have demonstrated their promise as nanofibers and have been used in various high-performance applications, including oil–water separation, bio-filtration, personal protective equipment, drug delivery, power storage, etc. where the smaller fiber diameter is highly valued.

There is a perceived dearth of review papers on polyolefins in the scientific literature, despite their rising demand. Brown and co-workers have completed a full review of melt-electrospun polyolefin nanofibers[18]. A thorough overview of the developments in melt-electrospun nanofibers and their potential uses was provided by Zhang et al. [19]. However, melt electrospinning of polyolefin nanofibers has been the subject of a few reviews, but the discussion about solution electrospinning for this material has not been investigated. This is a significant area for possible review studies since solution electrospinning has the potential to give distinct benefits in terms of fiber morphology control and the capacity to integrate diverse functionalities. So, a comprehensive review of developing advanced polyolefin nanofiber should discuss both melt and solution electrospinning and their benefits and drawbacks. Furthermore, numerous books on polyolefins have been published [20–23]; however, a comprehensive discussion of all classes, forms, and areas of application in a single frame is noticeably absent.

Polyolefins are an active area of research; nevertheless, there is a significant shortage of published comprehensive review papers. This discrepancy highlights the need for an up-to-date and in-depth overview of recent advancements and the state-of-the-art techniques in polyolefins. The objective of this review paper is to present a comprehensive analysis of two significant research advancements in the field of polyolefins: the synthesis and novel applications of polyolefin fiber and nanofiber. This review paper can fill a void in the literature by providing a comprehensive analysis of these critical areas. Besides, the major advancements and applications of polyolefins in various areas, including home, industry, automotive, agriculture, filtration, bio-medical, protective clothing, and power storage have been summarized in this study. Moreover, this review concludes with a concise overview of recent developments in polyolefin electrospinning and their potential implementation in the form of polyolefin nanofibers. A meticulous discussion of the challenges, potential remedies, and prospects in the electrospinning of polyolefins has also been delineated in the current review.

## Polyolefin fibers

Polyolefin fibers are primarily aliphatic polymeric hydrocarbons that are composed of at least 85% by mass of olefin units. The US Federal Trade Commission defined polyolefin fiber as “*A manufactured fiber in which the fiber-forming substance is any long chain synthetic polymer composed of at least 85% by weight of ethylene, propylene or other olefin units*” [24]. Among the polyolefin fibers, commercially PP is considered the most significant fiber, and to a lesser extent, polyethylene (PE). Polyolefin fibers are well-known in the field of textiles due to their inherent high tensile strength (both wet and dry) and hardness, good abrasion resistance, and excellent chemical and stain resistance properties [1]. Therefore, these fibers replaced both natural and other existing manmade fibers rapidly in various potential textile applications from the very beginning of their commercialization. In recent times, the development of advanced and innovative textile products based on polyolefin fibers has been eagerly welcomed by the majority of global consumers. The polyolefin fibers are mostly made of isotactic polypropylene, ultra-high molecular weight polyethylene, and high-density PE. In commercial textile applications, however, the significance of other alpha-olefin polymeric fibers compared to PP is very low. According to the report of the China Chemical Fiber Association, about 95% of polyolefin fibers are PP, including staple fibers and filament yarns, which fulfill at least 6% of global annual fiber production demand [25].

### Polyethylene fiber

Polyolefin monofilament extruded from low-density polyethylene (LDPE) was the first textile fiber developed in 1930s. However, these LDPE fibers gained limited attention initially for commercial purposes due to their coarseness and poor mechanical properties, as the fibers were derived from the low molecular mass polymer [26, 27]. Some high-density polyethylene (HDPE) fibers and ribbons were commercialized at that time namely, “Courlene” and “Reevon” [28]. However, the expedient applications of HDPE monofilaments as a general textile fiber were restricted due to their low resilience, poor softening range, and high deformation against stress (creep). Nevertheless, these coarse monofilament/ribbon-like polyolefin fibers were widely utilized as outdoor fabrics, protective clothing, filter fabrics, ropes, webbing, nets, cordage, conveyor belting, and seamless medical tubes [29, 30]. Linear low-density polyethylene (LLDPE) monofilaments, ribbons, and slit-film weaving tape were also introduced in a small quantity for non-textile applications. Since various types of ropes, nets, and cordages that were generally manufactured from natural fibers were replaced by HDPE monofilament, and its weaving tapes became popular for fabricating diverse industrial fabrics like tarpaulins, shade mesh fabrics, and geotextile membranes.

Although the theoretical evidence of high modulus polyethylene fibers has been available since the 1930s, the ultimate outcomes were found in the mid-1980s, when the commercial ultra-high molecular weight polyethylene (UHMWPE) fibers were developed with suitable spinning techniques [31, 32]. Research and development of high-performance UHMWPE filament fibers have continued on many fronts. In 1970, high-modulus polyethylene fibers were commercialized by Ward et al. [33] and received their license as Tenfor and Certran. However, during the production, the high melt viscosity and intermolecular entanglement of UHMWPE significantly hampered the extrusion and subsequent drawing for the orientation of fibers. Therefore, the gel spinning process was introduced [34], which solved all of the deficiencies, and successfully developed high-strength, high-modulus polyethylene fibers in 1981. The revolution in the PE fiber industry was then initiated, and to date, the UHMWPE fibers are commercially renowned as Dyneema and Spectra in the global market. The development of ultra-high modulus fibers and yarns like Dyneema, Spectra, and Tensylon extended the scope for using polyolefins in a high strength demanding applications, including antiballistic fabrics, deep-sea mooring ropes and braids, and highly efficient medical devices [35]. The common trade names of UHMWPE include, ${{\text{Dyneema}}}^{{\circledR }}$, ${\mathrm{Dyneema Purity}}^{{\circledR }}$ by DSM, ${{\text{Spectra}}}^{{\circledR }}$ by Honeywell, and ${{\text{IZINAS}}}^{{\circledR }}$ by Toyobo.

**Fig. 1:**
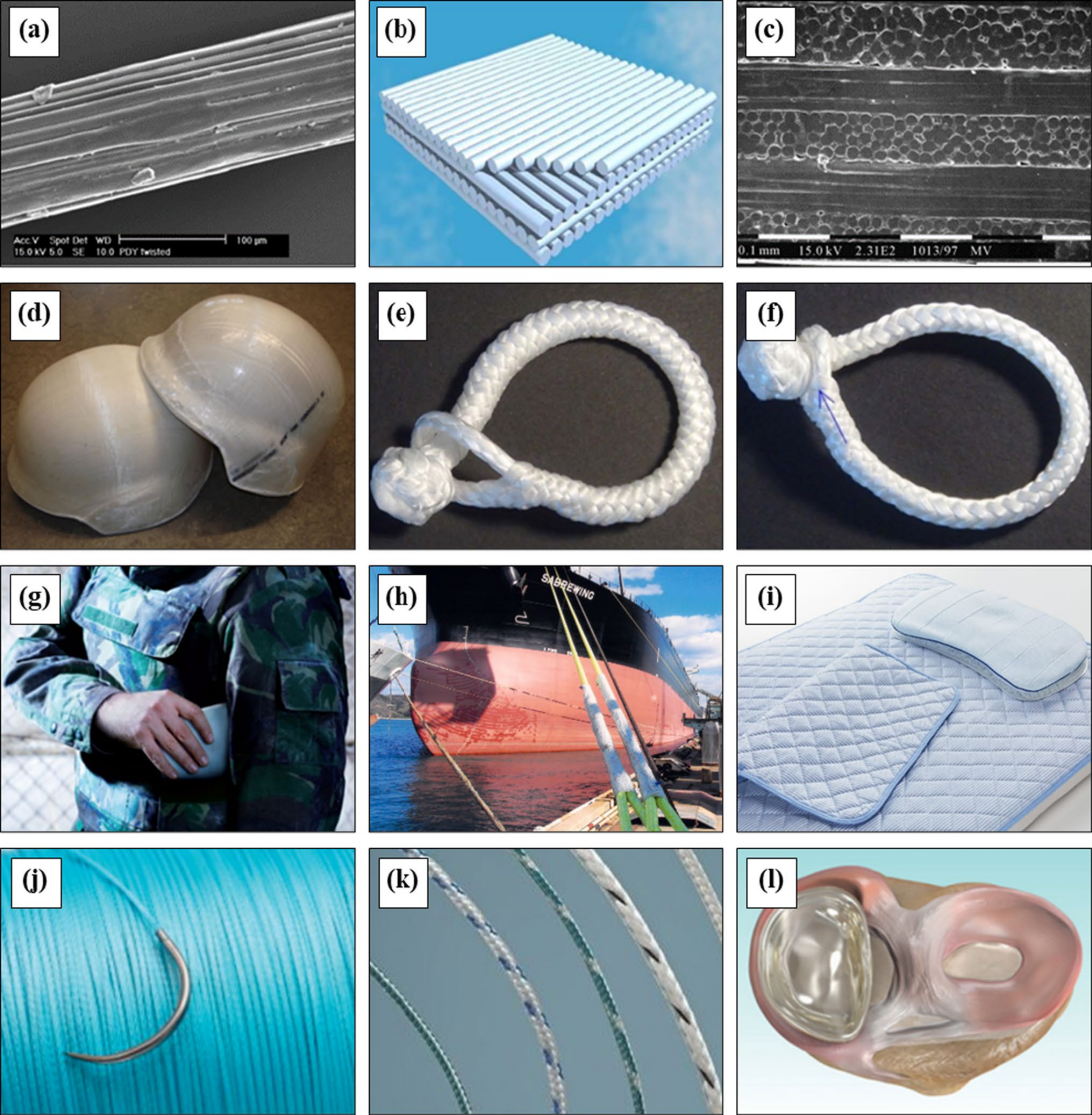
**a** SEM image of a ${{\text{Dyneema}}}^{{\circledR }}$ monofilament, **b** Illustration of fiber cross plies for armor, **c** SEM micrograph of a cross section of ${{\text{Dyneema}}}^{{\circledR }}$ monofilament [38], **d** Two helmet shells made by creep-forming of gelspun UHMWPE fibers [36], **e** Soft shackle made with UHMWPE in open condition, **f** In closed condition [38]. **g** Body armor made by UHMWPE fiber, **h** Mooring rope of ${{\text{IZINAS}}}^{{\circledR }}$ used for ultra-large ships. *Courtesy of Toyobo, Japan*, **i** ${{\text{ICEMAX}}}^{{\circledR }}$ fabric made by combining ${{\text{IZINAS}}}^{{\circledR }}$ with special fibers for high sensation of coldness. *Courtesy of Toyobo, Japan*, **j**, **k** Braided surgical suture from blue Dyneema Purity® fibers. Courtesy of DSM, Netherlands, **l** Dyneema Purity® fiber reinforced implant used for meniscus replacement. *Courtesy of Active Implants*

The SEM illustration of commercial ${{\text{Dyneema}}}^{{\circledR }}$ fiber is shown in Fig. 1a. The gel-spun UHMWPE fibers are lightweight with zero moisture regain and have high strength, which is generally considered as a strong competitor of carbon and kevlar fibers in the application field of protective clothing and high-performance sails as shown in Fig. 1g [36]. Fibrous armor plates are more promising for bulletproof vests where the perpendicular direction of fiber arrangement can perfectly resist fast projectiles. Indeed, ${{\text{Dyneema}}}^{{\circledR }}$ fibers have many successful applications as a typical armor material. Using the easy creeping property of gel-spun UHMWPE, the helmet shell has been made by cross-ply fiber arrangement and creep-forming process at about 130 ℃ as shown in Fig. 1b, c, d. Here, the low melting temperature drawbacks of UHMWPE are turned into an advantage as easy shaping, fusing, and simultaneous cutting of edges [37, 38]. The chemical and abrasion resistance makes the ropes made of these fibers more attractive and a good alternative to cables and metal wires in corrosive environments [39, 40]. Metal shackles are widely used as connections and load-bearing materials; however, they are hard, heavy, permanent, and environmentally hazardous. Soft shackles made from gel-spun UHMWPE fibers are a potential alternative for their high tensile strength, extreme damage tolerance even light and slippery properties [38]. Moreover, such soft shackles can easily be opened even after a heavy loading, as shown in Fig. 1e, f. Mooring ropes of ${{\text{IZINAS}}}^{{\circledR }}$ are much lighter than wire, safer to handle, and can be used without greasing, making them ideal for use on ultra-large vessels like ore carriers, crude oil tankers, and LNG tankers as shown in Fig. 1h. The thermal conductivity coefficient of ${{\text{IZINAS}}}^{{\circledR }}$ is approximately four times higher than that of standard materials. In laboratory tests, the ${{\text{ICEMAX}}}^{{\circledR }}$ fabric formed by combining ${{\text{IZINAS}}}^{{\circledR }}$ with special fibers provides twice the cooling sensation of traditional materials as shown in Fig. 1i [41].

The medical grade gel-spun UHMWPE fiber is known as Dyneema Purity® as shown in Fig. 1J, k, l. This fiber is considered as a gold material for medical applications because of their fitness, high strength, abrasion resistance, low elongation and good compatibility with human body over the longer lifespan [42]. Very common and recent surgical applications of Dyneema Purity® fiber includes surgical sutures, surgery of the spine, arthroscopic procedures, ligament repair, trauma, and implant reinforcement [38, 43]. 

### Polypropylene fiber

Polypropylene was first discovered in 1951 and commercially appeared as a superior plastic material in 1957. The first PP monofilaments for textiles, however, began to appear in the market in the late 1950s. In the early 1960s, PP in the form of staple fibers and multifilament was produced in small quantities in Italy, UK, and USA for use in textiles [27, 44]. The first commercial multifilament and staple fibers from isotactic polypropylene were melt-spun employing similar equipment and techniques that were commonly used for polyamide and/or polyester fibers. The innovative use of PP in textiles was started when continuous PP tape yarns (ribbon) with high strength were invented from the extruded film in the late 1960s [22]. In the early 1970s, the revolutionary growth of PP tape and nonwoven fabrics ultimately eliminated the use of traditional jute yarn and fabrics and supplanted it with the new polyolefins in the textile industry. Consequently, the most renowned jute carpet backing and sacking cloth was substituted by PP tape fabrics. Moreover, the cords, rope, and twines prepared from natural fiber spun yarn was replaced by PP tape yarns in many cases. This revolution of polyolefin tape yarns crossed the border of Europe and North America within a short period and spread to the whole world.

However, the PP tape yarns prepared from the extruded film were not considered as textile fiber or yarn due to their irregular cross-section, size and shape, less luster, and rough textures. Subsequently, the continuous filament tows were prepared from polymer melt of solid PP granules using a spinneret plate with cooling air stream [45]. “James Mackie & Sons Ltd” company introduced a system to prepared PP staple fiber and commercialized rapidly, through which in-house PP fiber supply was easily available globally to the spinners [46, 47]. The industrialization of PP textile products through weaving, spinning, and carpet manufacturing, was initiated in the mid-1970s and well established by the late 1970s. During the 1980s, the industrial expansion of PP was stimulated by a wide range of innovative products utilizing industry-oriented innovative research and development [22].

The PP fiber available in a variety of renowned textile forms is mainly prepared from isotactic PP. Currently, PP fulfills approximately 6% of global annual fiber production demand in a weight of 4.3 million tons; mostly filament yarns and a lesser amount of staple fibers [25]. Textile fibers made of PP, in particular, have been progressively well-known to consumers and manufacturers because of their huge applications in carpets and nonwovens. Replacing nylon and jute, PP aggressively captured the largest market of carpets and nonwovens for use in a large variety of products, including wet wipes, baby diapers, hygiene fabrics, and adult incontinence fabrics. PP fibers also have prominent textile applications, such as disposable hospital equipment, geotextiles, automotive fabrics, and industrial wipes [48].

### Polyolefin blend fiber

Polyolefin blend fibers are another types of fibrous material prepared by adding another polymer to polyolefin solution before spinning in order to enhance or improve some aspects of targeted functionalities, physical characteristics, degradability, and applicability [49]. Polymers having miscibility with polyolefin can easily be employed to develop blend fibers with desired characteristics, but in practice, a few miscible polymer pairs are available for such purposes. Hence, the addition of compatibilizer for immiscible polymers, as a third particle, is considered the best technique for fabricating the alloy fibers with enhanced physical properties [50]. The melt blending a mix of ‘additive masterbatches’ to the main polymer is a long-established and renowned method for the manufacturing of polyolefin textile materials. To prevent the longitudinal fibrillation of PP slit film yarns, 2–3% PE is commonly added from long time ago. The addition of 20% polyethylene terephthalate (PET) to PP has enhanced the tensile properties of PP/PET alloy tapes which are more prominent as carpet backing, ropes, and engineering fabrics [51, 52]. In 2007, a Chinese patent first demonstrated PP/PET blend fibers with a 30% increment in tensile strength of alloy fibers compared to pure PP fibers [53]. Likewise, melt blend fibers of 92% PP and 8% atactic polystyrene significantly improve the creep resistance and rigidity of the developed fibers [54]. Another potential PP/polystyrene melt blend fibers with only 5.0% polystyrene demonstrated the reduction of undrawn fiber crystallinity, which was employed to assist the texturizing of PP multifilament yarns [55]. A variety of alloy fibers was also drawn using different ratios of PP/liquid crystal polymers [56], PP/HDPE [57], iPP/aPP [58], and iPP/polylactic acid (PLA) [59] blends for enhancing mechanical properties and biodegradability of fibers.

## Polyolefin nanofibers

**Fig. 2:**
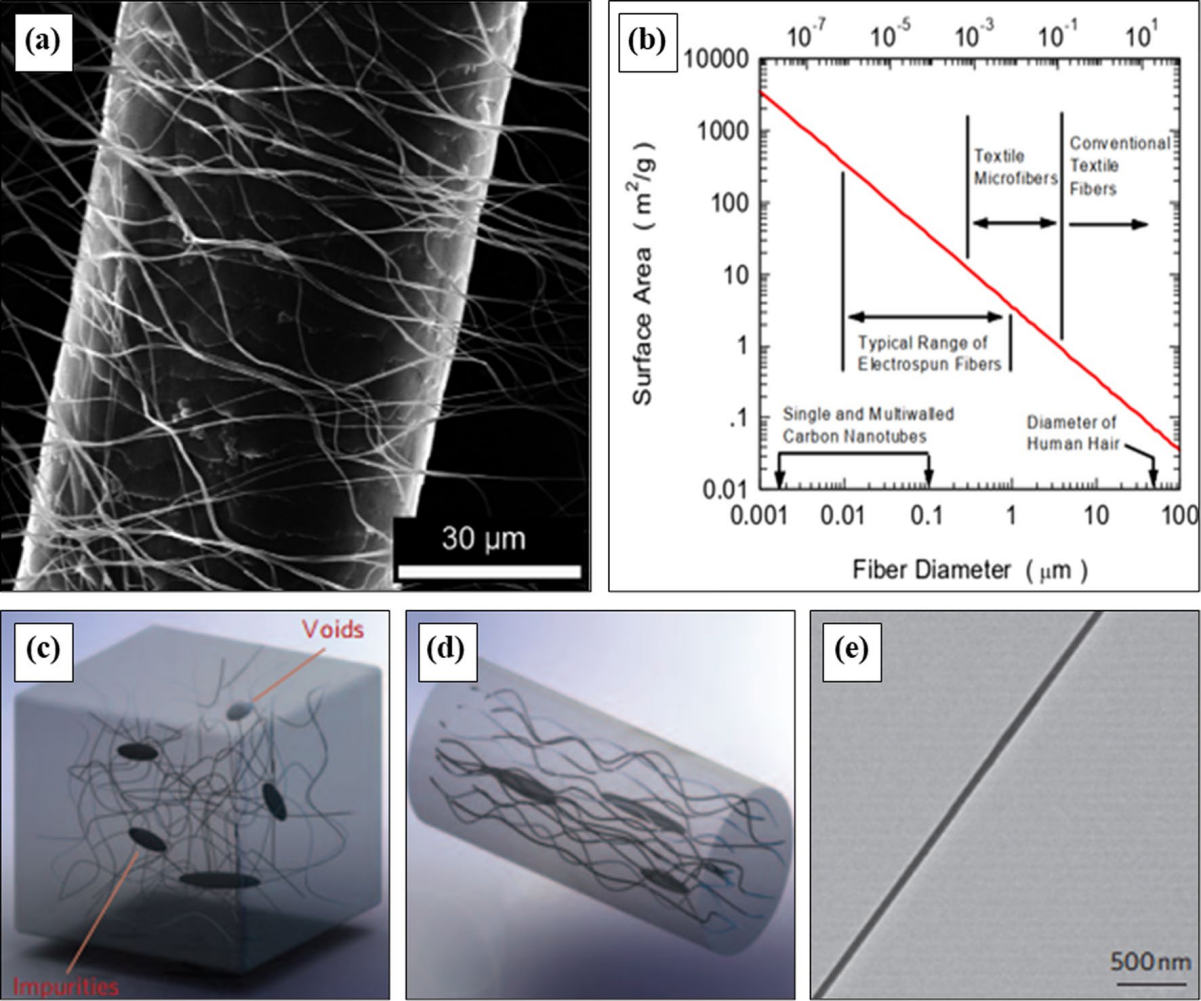
**a** SEM image of a human hair surrounded by electrospun nanofibers [61], **b** relation between fiber diameter and surface area [68], illustration of (**c**) bulk polyethylene containing chain ends, entanglements, voids, and defects, **d** stretched polyethylene microfiber, **e** TEM image of a perfectly aligned polyethylene nanofiber [62]

The term ‘nanofibers’ refers to fibers having diameters varying from a few tens of nanometers up to several hundred nanometers, but generally less than one micrometer [60]. Electrospun nanofibers are much finer than human hair, the visual comparison is shown in Fig. 2a. Some practical examples of fiber in the micro or nanoscale dimension can make that visual comparison easy to understand. One gram fiber with a diameter of 10 µm, contains at least 13 km in length and a specific surface area of 0.4 m^2^ g^−1^. Instead, the same amount (1 g) of fiber with 100 nm in diameter comprises the total fiber length around 130,000 km and the specific surface area of 40 m^2^ g^−1^. Schreuder-Gibson et al. [61] reported a relation between nylon 6, 6 fiber diameter, and surface area including typical diameter ranges of various application areas, as shown in Fig. 2b. In fiber technology, denier is the unit of synthetic fiber fineness, which denotes the mass of 9 km fiber. For the first case, the fiber fineness is 0.69 denier, while in the second case, it is about 0.69 × 10^–4^ denier. Polyolefins have some outstanding characteristics, including high chemical resistance, ease of processing, near-zero moisture absorption, electrical properties, low friction coefficient, low cost, and abrasion resistance, which ensure its largest volume in the world polymer consumption [10–12, 62, 63]. However, the comparative low strength of bulk polyolefin greatly hinders its functionality [62]. Instead, a significant enrichment of the mechanical and thermal resistivity of polyolefin has been reported by the proper alignment of its polymer chains [64, 65]. Therefore, the stretching of olefin polymer from bulk into thin films, or micro- or nanofibers, improves the polymer chain arrangement and results in improved mechanical and physical properties [66, 67]. Moreover, only the fiber diameter change from micrometer to nanometer range resulted in a rapid enhancement of the specific surface area, surface functionalities, cohesiveness, and even mechanical properties [13–16].

**Fig. 3:**
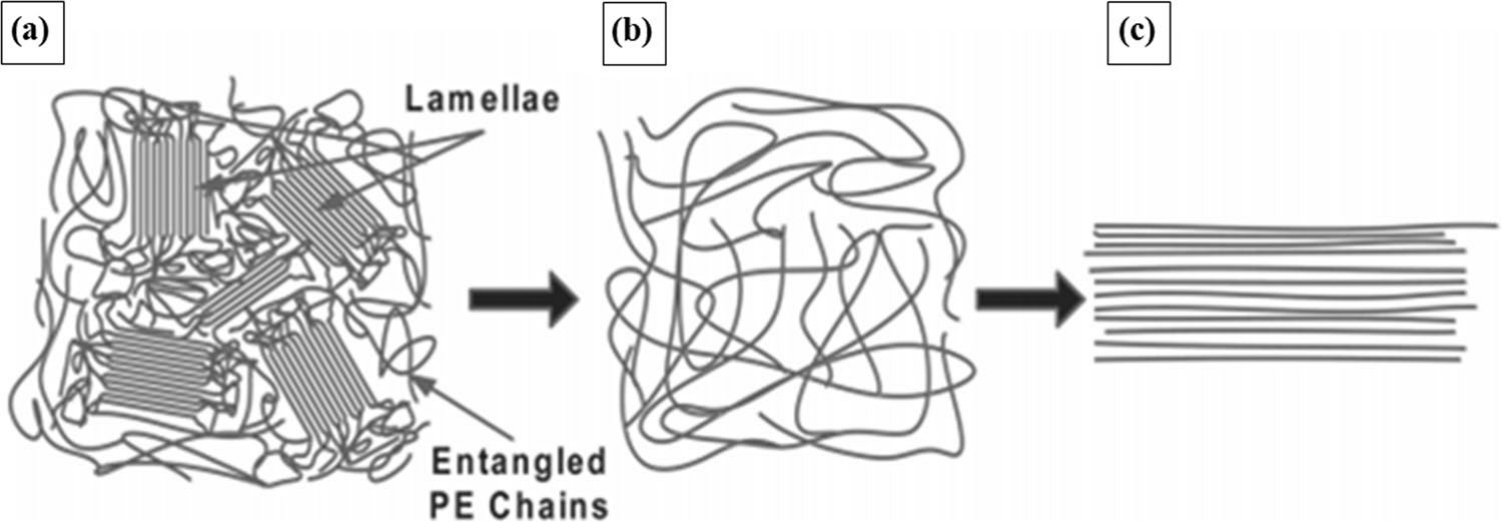
**a** Bulk PE with amorphous and crystalline regions in gel, **b** Randomly entangled PE chains in Decalin solution gel at high temperature, **c** PE nanofiber with well-aligned polymer chains [70]

The low strength and stiffness of bulk polymers are due to the chain ends, entanglements, voids, impurities, and so on [69]. The well alignment of molecular chain and stretching of bulk polymer by mechanical drawing or electrostatic force minimized that defects, which results in enriched mechanical and physical properties of polymeric fiber [62, 70, 71], as shown in Fig. 2c, d, e. In nanofiber the amorphous region is unlikely to present, thus a subsequent lower concentration of defects can exist in a nanofiber than microfiber. Li et al. [70] prepared an extremely highly crystalline (> 90%) PE nanofiber using a gel-based electrospinning method equipped with two-stage heating and drawing. They demonstrated the molecular states of PE during nanofiber preparation, are shown in Fig. 3. At room temperature, both ‘lamellae’ (locally folded PE chains) and other entangled PE chains are randomly distributed in the gel (Fig. 3a). After heating at 120 ºC, all PE molecular chains are free to move (Fig. 3b) and in nanofiber, PE chains are placed in a good alignment without any defects (Fig. 3c).

Consequently, PE nanofiber with the highest crystallinity above 90% and Young’s modulus of 312 GPa was found [70]. Ma et al. [71] also explained the similar alignment process of PE chains during solution electrospinning at 120 ºC. They observed the improved crystallinity and 20 times higher thermal conductivity of electrospun PE nanofiber than bulk polymer due to their highly oriented polymer chains. Shen et al. [62] prepared PE nanofiber with a diameter of only 50 to 500 nm using a two-step drawing process and reported a high thermal conductivity as ~ 104 Wm^−1^ K^−1^. While the thermal conductivity of bulk polymers is only 0.1 Wm^−1^ K^−1^ [72] and for commercial PE microfibers with 10 to 30 µm in diameter is 30–40 Wm^−1^ K^−1^ [73, 74]. The high surface to volume ratio of nanofiber ensures excellent surface covering that acts as a potential barrier against targeted fine particles and microorganisms during filtration or separation [17]. Owing to these, polyolefins evidenced their potentiality as a nanofiber and emerged in a wide area of high-performance applications, such as filtration, protective clothing, bio-medical, and power storage, where the reduced fiber diameter was highly appreciated.

## Preparation of polyolefin nanofibers

**Fig. 4:**
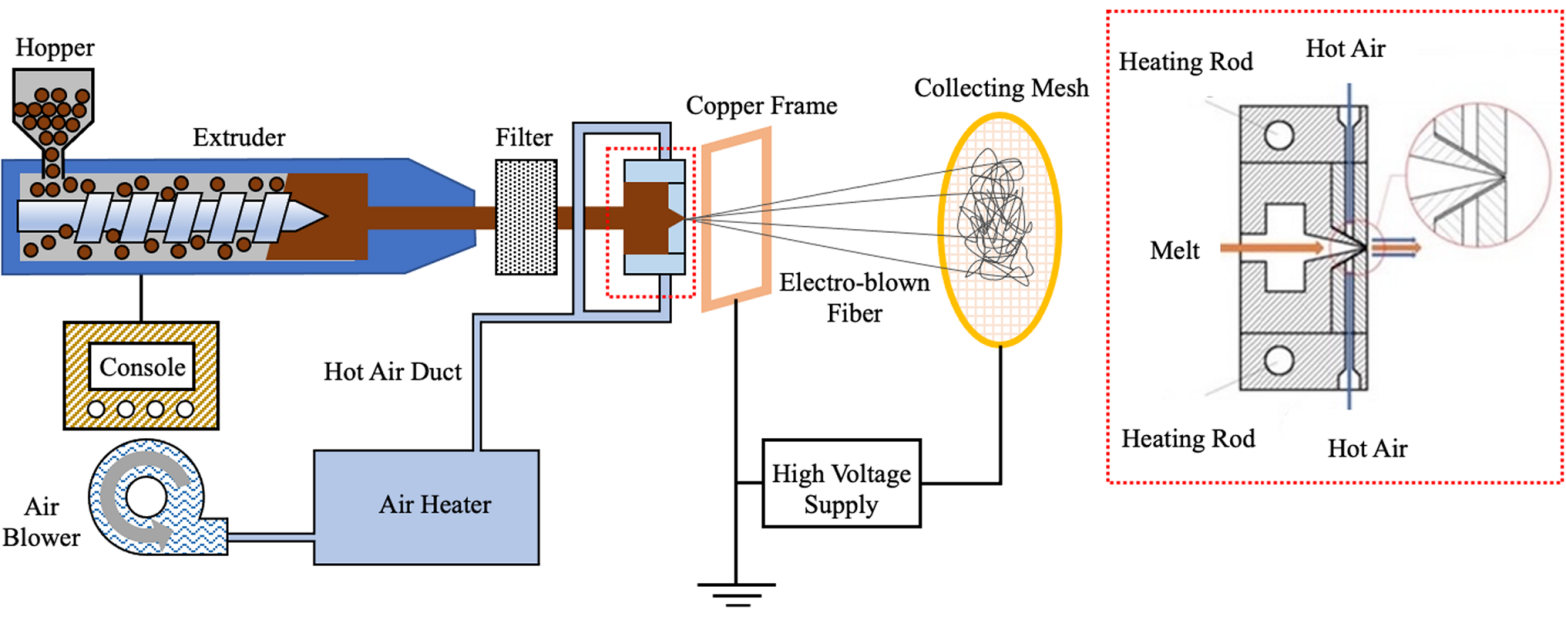
The schematic illustration of the electro-blowing process

Electrospinning is the simplest, efficient, and more prominent technique to prepare nanofibers [18, 75]. Although, many other processes including melt blowing [76–80], centrifugal spinning [81, 82], template synthesis [83–85], and high-temperature drawing [62], [70] are involved to prepare polyolefin fibers with a nanoscale dimension. In the melt blowing process, polymer melts are extruded through a spinneret (die) with a high-speed air/gas stream often heated and the fibers are deposited to the collector as instantly spun-bonded nonwoven mats [86, 87]. The pressurized jet blowing instigates the quick super drawing of ejected melt fiber and reduces the diameter from micro to sub-micron or even nanometer range [88, 89]. PP nanofiber with diameters ranging from 75 to 375 nm has been prepared using this technique [77]. Recently, the highest volume of the nonwoven web with ultrafine polymeric fibers is yielded from the melt blowing process. Pu et al. [90] prepared PP nanofiber incorporating electric field in melt-blowing process termed as “electro-blowing” [91], shown in Fig. 4. Furthermore, fibers with a diameter below 500 nm can only be achieved for high melt flow index PP types using melt blowing [79].

Centrifugal or force spinning is another renowned method for high-speed production of commercial polyolefin nonwoven mat. However, the ultra-speed filament injection and instant spun bonding of these methods greatly hinder the aligned individual fiber preparation with a uniform diameter [92]. A membrane having cylindrical/hexagonal straight and separated mesopores with a monodisperse diameter in the nanometer scale is used as a template to prepare polymeric nanofibers [84]. This template synthesis method is well known to prepare nanofiber with a too low range in diameter. High molecular weight PE nanofiber has been prepared with only 30–50 nm in diameter using this technique [93]. Nevertheless, the fiber length is strictly limited to the length of pores placed in the template is generally shorter than 60 μm [83]. High molecular weight PE nanofiber also has been reported below 100 nm in diameter using high-temperature drawing method but its non-uniform straining along the fiber length extends the ultimate fiber diameter ranges [62, 70]. The electrostatic spinning eliminates the issues regarding excessive drawing speed, uneven mechanical straining, fiber length limitation and ensured the high productivity of continuous or nonwoven web of polyolefin fibers with a wide range of diameters from < 50 nm to 500 µm [18, 94, 95]. Therefore, electrospinning is considered to be the more potential technique for the mass production of polyolefin nanofibers to meet up a vast area of potential application.

## Electrospinning of polyolefins

**Fig. 5:**
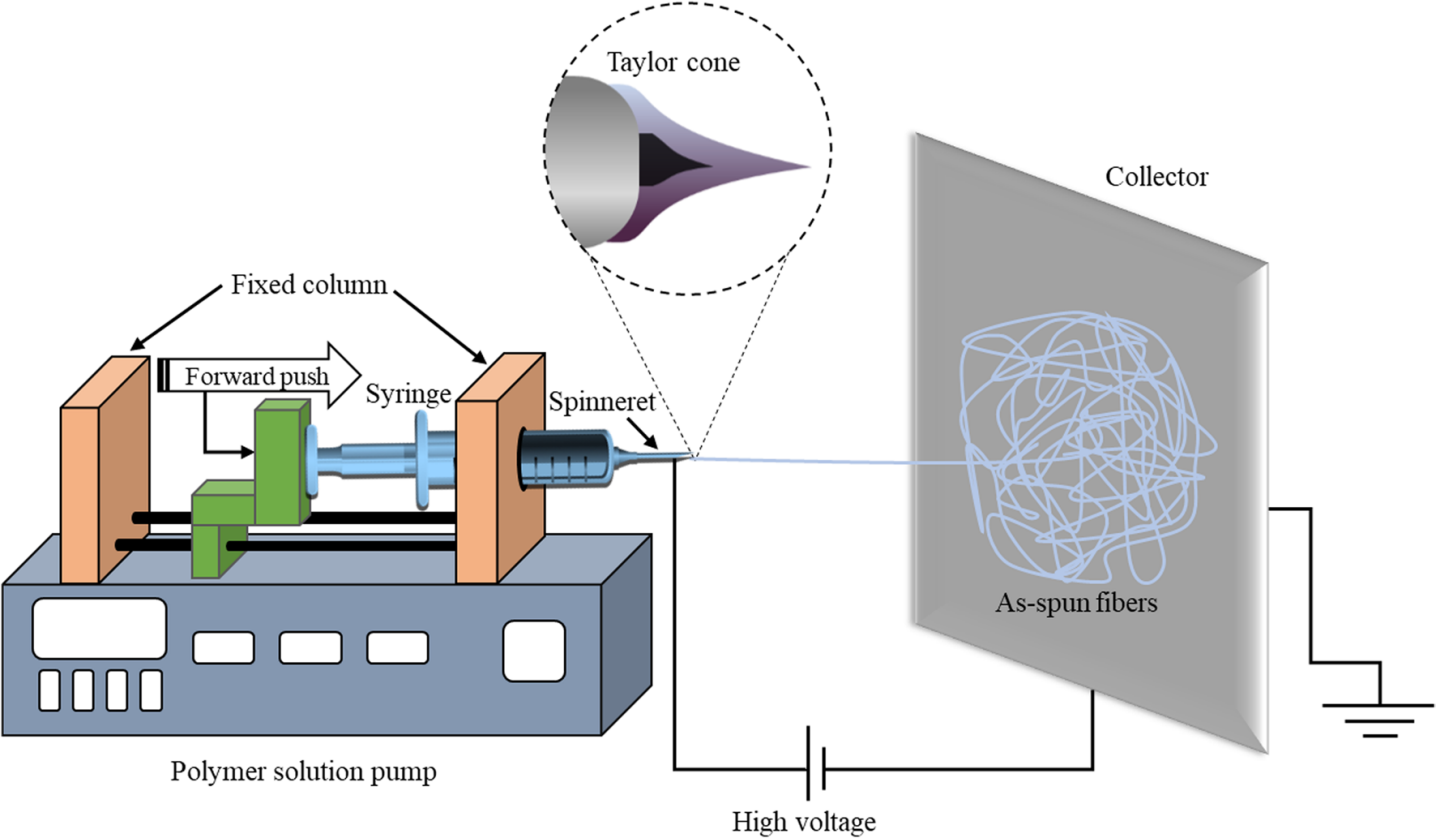
Schematic diagram of a basic electrospinning process

The term ‘‘electrospinning’’, derived from “electrostatic spinning’’, was first used recently in around 1994, although its fundamental idea was developed more than 60 years ago [13]. Electrospinning is a spinning process of ultra-fine fiber based on an electrohydrodynamic (EHD) phenomenon that uses electrostatic force to draw continuous fibers, in the form of a liquid jet, from a polymer solution or melt [96]. A schematic diagram of the basic electrospinning process of polymeric nanofibers is shown in Fig. 5. The electrospinning process consists of three components: a capillary tube with a pipette or needle, a high voltage supplier, and a grounded metal collecting screen. Although, the needless electrospinning eliminates the capillary tube where materials are directly supplied in the form of a pellet, rod, or sheet. Generally, the polymer solution or melt is poured into the capillary tube and the high voltage is supplied by an electrode adjacent to the pipette or needle. A well-controlled continuous supply of polymer solution or melt through the tip of the electroactive pipette or needle results in an electrically charged jet. The high electrostatic force between the electrode and grounded screen drag this polymer jet to the collector as an interconnected web of ultra-fine fibers. The polymer solution fluid is normally placed to the end of the needle and held by its surface tension [97]. The electric field subject to the polymer droplet and due to the mutual charge attraction, the counter electrode creates a force against its surface tension. The progression of the electric field intensity elongates the hemispherical surface of the polymer droplet and forms a conical shape generally referred as the Taylor cone [98]. The further intensity increment advances the repulsive electrostatic force over the surface tension of the polymer droplet that ejects the charged fluid jet from the tip of the Taylor cone. The discharged fluid jet passes through a process of elongation and EHD instability, which results in an ultra-thin and long jet. Meanwhile, the thin jet evaporates or solidifies during its travel into the air and is collected as a fine polymeric fiber by the metal collector. Electrospinning is a renowned process for manufacturing ultra-thin nanofibers from around fifty more varieties of polymer with a wide range of diameters from < 3 nm to over 1 µm [13].

**Fig. 6:**
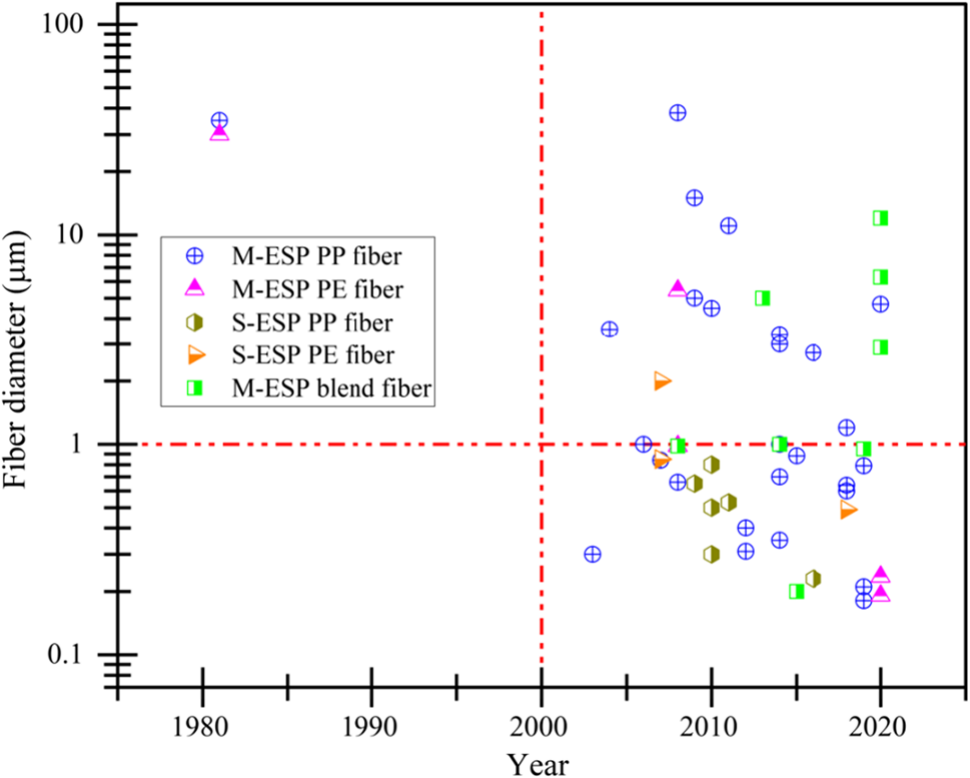
Recent reports on electrospun polyolefin fibers

Figure 6 sums up the recently published research articles regarding electrospun polyolefin fiber preparation from the beginning considering the fiber diameter against the publication year. The story of electrospun polyolefin fiber preparation started in 1981, causing a long research gap up to 2003 due to the solvent dependency, less interest for coarser fiber production, and the complexities of spinning apparatus [99, 100]. Solution electrospinning is more advantageous to prepare polyolefins nanofiber below 1 micron in diameter, however, the unavailability of proper solvent at room temperatures largely hinders its popularity. M-ESP is considered an effective alternative for the preparation of polyolefin nanofibers due to its solvent independency and environmentally friendly approaches, nevertheless, the high viscosity and non-polarity of polymer melt pose difficulties to obtain nanoscale fiber dimension. The recent techniques such as the use of high MFI polymers [90, 101], viscosity reducing additives [102], polarity improving salts [14, 103], and blends [104] are found more successful to minimize the diameter of melt-electrospun polyolefin fiber. Thus, the potentiality of electrospinning is increasing day by day that augments the existing fiber technologies and ensures the niches in industrial sector of new high-performance polyolefin nanofibers.

### Polyethylene nanofiber

**Fig. 7:**
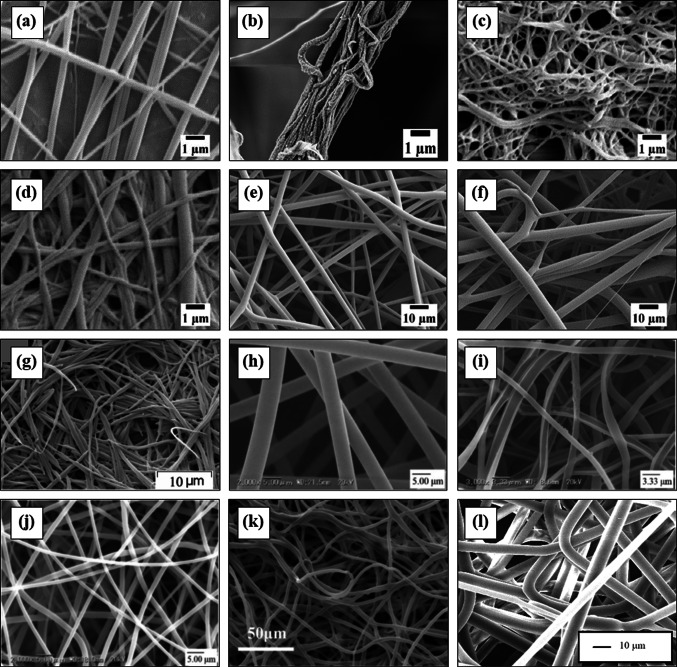
SEM images of melt-electrospun polyolefin nanofibers from some recent investigations, **a** PP [113] permission with John Wiley & Sons, Inc., **b** PP [114] permission with John Wiley & Sons, Inc., **c** PE [110] permission with John Wiley & Sons, Inc., **d** PE [109], **e** PP/PVB blend [113] permission with John Wiley & Sons, Inc., and **f** PE/PVB blend [113], **g** PP from CAB/iPP [115], **h** PP [104], **i** PP from PP/EVOH film [104], **j** PP from PP/EVOH/PP film [104], **k** SAN/iPP [116] blend; **l** PP electrospun in vacuum [100]

The electrostatic spinning of PE from polymer melt started together with PP in 1981 but a few research articles were found to date [99]. Among the PE grades, ultra-high molecular weight polyethylene (UHMWPE) attracts more attention to researchers and industrialists for a unique combination of its properties including outstanding chemical and abrasion resistance, lubricity, and unmatched toughness [1, 39, 105]. However, electrospinning of UHMWPE with a nanoscale dimension is very tough due to its too high viscosity and non-polarity even using the solution electrostatic method. Table 1 lists the published articles to date on electrospun PE fibers indicating the key particulars. Solution-gel (sol–gel) method is more renowned for preparing super-strong high-performance commercial UHMWPE fiber namely${{\text{Dyneema}}}^{{\circledR }}$,${{\text{Spectra}}}^{{\circledR }}$, and${{\text{IZINAS}}}^{{\circledR }}$. Park and Rutledge combined both sol–gel and electrospinning techniques and prepared UHMWPE nanofiber with 490 nm in diameter. They employed t-BAB (tetrabutylammonium bromide) with a polymer solution, which improves the polarity of PE and minimizes the electrospun fiber diameter greatly. Moreover, they reported an unparalleled combination of mechanical properties including Young’s modulus, tensile strength, and toughness [106]. Compared to solution electrospinning, however, there have been few attempts to produce PE nanofibers using melt electrospinning (M-ESP) technique. SEM images of melt electrospun PE nanofibers are shown in the Fig. 7c, d. Deng et al. [107] demonstrated the development of PE nanofiber with a diameter of 5 µm using a simple melt electrospinning (M-ESP) device. Yang et al. developed an M-ESP device integrated with a heated oil-circulated polymer melting system and prepared LDPE nanofibers with a diameter range from 1.2 µm to 50 µm [108]. Two recent investigations have reported melt-electrospun LDPE nanofibers only 235 nm and 191 nm in diameter using polyvinyl butyral (PVB) and ethylene-co-vinyl alcohol (EVOH), respectively as a blend component [109, 110].

**Table 1:** Electrospinning of PE with processing technique and resultant fiber diameters

PE types	Material property (Mw in g/mol, MFR in g/10 min	Processing technique	Used solvents/additives/ Blends	Heating temperature	High voltage supply (kV)	Fiber diameter (µm)	References
LLDPE	Mw 190,000	S-ESP	P-xylene, t-BAB	Infrared heater 105–110 °C	8 (Needle + ve)	2 to7	[111]
UHMWPE	Mw 6,000,000	S-ESP	P-xylene, cyclohexanone	Silicon oil bath/reservoir 100–160^O^C, needle 90–170 °C	7–25 (Needle + ve)	0.85 to 1.3	[112]
UHMWPE	Mw 4500	S-ESP	P-xylene, t-BAB	Glass syringe 170 °C	15–20 (Needle + ve), 10–15 (Collector -ve)	0.49	[106]
UHMWPE	Mw 3–6 × 10^6^	S-ESP	P-xylene, cyclohexanone	Radiant heater/glass syringe 120 °C	9–52 (Needle + ve)	–	[71]
HDPE	MFR 2	M-ESP	Paraffin	Electrically heated Al jacket 200–220 °C	10–23	30 to 135	[99]
LDPE	MFR 2	M-ESP	–	Electrical heating/piston 315–355 °C	30–60 (Collector)	5.44 to 32.37	[107]
LDPE		M-ESP	PE mono alcohol, PE wax, Oxidized PE wax	Heated silicon oil circulation/syringe 190–230 °C	17–23	1.25 to 50	[108]
LDPE	MFR 13	M-ESP	PVB	CO_2_ laser	40 (Electrode + ve)	0.235	[109]
LDPE	MFR 13, 23, 45, 70, & 145	M-ESP	EVOH	CO_2_ laser	40 (Electrode + ve)	0.191	[110]

t-BAB; tetrabutylammonium bromide

### Polypropylene nanofiber

PP, which is impossible to dissolve in any solvent at room temperature, was one of the first thermoplastic polymers to be melt electrospun [99]. PP is the lightest of polymeric fibers, has acquired significant attraction to textile technologies for its strength, flexibility, and excellent chemical resistance. A comprehensive list of all electrospun PP fibers to date including types, techniques, and parameters is presented in Table 2. Where the articles are arranged according to the descending order of developed fiber diameter starting from solution electrospinning. The diameter of electrospun fibers is greatly dominated the ultimate functionality and applicability. Reduced polymeric fiber diameter is always appreciable due to the augmentation of specific surface area, which ensured the progressive surface covering against micro to nano-sized particles during filtration and separation. Similarly, solution electrospinning is more potential to prepare PP nanofiber with very less diameter. The syndiotactic PP is found more preferable for solution electrospinning due to its low melting point, although, around 90–95% commercial PP is isotactic. Lee et al. [117] first used the solution electrospinning method to prepare PP nanofiber with a diameter of 0.65 microns in 2009. They employed “Cyclohexane” as an additive in order to improve the electrical conductivity of the non-polar PP solution that resulted in the reduced fiber diameter. Later, Maeda et al. [77] fabricated solution electrospun syndiotactic PP nanofiber with a diameter of only 230 nm and suggested that methyl-cyclohexane is more effective solvent. Still, M-ESP is the best and widely used technique to prepare PP nanofiber due to the unavailability and toxicity of solvents. The high viscosity, low conductivity, and exit swelling of the polymer melt in M-ESP are considered as strong obstacles for obtaining PP fiber in a suitable nanoscale dimension. Various attempts have been made to overcome this barrier, where the addition of salts to PP as an electrical conductivity improver is found more fruitful [14, 103]. SEM images of melt electrospun PP nanofibers are shown in the Fig. 7a, b, g, h, i, j, l. Li et al. [118] introduced a needleless M-ESP device indicating the high productivity and employed high MFR (2000 g/10 min) PP but could not produce fiber diameter below 1 µm. Rangkupan and Reneker reported the first melt-electrospun PP nanofiber less than 1 µm in diameter, where the electrospinning was carried out in vacuume [100]. Dalton et al. [102] were able to reduce melt electrospun PP fiber diameter from 35 to 0.840 µm by applying “Irgatec” as a viscosity-reducing additive. Hollow PP nanofiber was manufactured with an average diameter of 640 nm from PP/EVOH/PP three-layer films using a line-like CO_2_ laser-beam M-ESP system [104].

**Table 2:** Electrospinning of PP with processing technique and resultant fiber diameters

PP types	Material property (Mw in g/mol, MFR in g/10 min	Processing technique	Used solvents/additives/blends	Heating temperature	High voltage supply (kV)	Fiber diameter (µm)	References
Isotactic PP	Mw 129,000	S-ESP	Decalin with 0.5% antioxidant	Electrical heated chamber/syringe 130 °C , needle 140–170 °C	3–8 (Needle + ve) 12 (Collector -ve)	0.8 to 9.6	[119]
Syndiotactic PP	Mw 127,000 MFR: 4.5	S-ESP	Cyclohexane, acetone, and DMF	Room temperature	10 (Needle + ve)	0.65	[117]
Syndiotactic PP	Mw 127,000 MFR: 4.5	S-ESP	Cyclohexane, acetone, and DMF	Glass syringe, metal tip 40 °C	10 (Needle + ve)	0.53 to 0.76	[120]
PP with 1-octene comonomer	Mw 506,700	S-ESP	Cyclohexane, acetone, and DMF	Room temperature	10–20 (Needle + ve)	0.50 to 2	[121]
Isotactic PP	Mw 280,000 MFR 3.0	S-ESP	o-DCB/ Bu4NClO4	Syringe/heated oil circulation 150 °C , needle tip/laser 120 °C	6–10 (Needle + ve)	0.3 to 1.2	[122]
Syndiotactic PP	Mw 174,000	S-ESP	methyl-cyclohexane	Room temperature	10 (Needle + ve)	0.23	[123]
Isotactic PP	Mn 5000	M-ESP	–	Cartridge heater; 230 °C	20–30 (Collector)	-	[124]
PP monofilament	–	M-ESP	–	CO_2_ laser; 5 W	20 (Nozzle + ve)	38	[125]
Isotactic PP	MFR 0.5	M-ESP	–	Electrically heated Al jacket; 220–240 °C	10–23	35 to 121	[99]
Isotactic PP	MFR 900 MFR 1500	M-ESP	–	Ceramic heater around syringe; 330 to 410 °C	35	15 to 25	[115]
PP	MFR 3.4	M-ESP	–	Extruder; spinneret 300 °C	25–35 (Collector + ve)	11.05 to 23.17	[126]
Isotactic PP Atactic PP	Mw 205,000, 12,000 Mw 19,600	M-ESP	Isotactic/atactic PP blends	Band heater around stainless syringe; 180–200 °C	12 (Collector + ve)	5 to 60	[127]
PP	MFR 3.4, 25 & 450	M-ESP	–	Extruder; spinneret 300 °C	25–35 (Collector + ve)	4.8	[128]
PP	MFR 1100	M-ESP	PP master batch	Heating coil/master batch 190 °C	27 (Needle + ve)	4.68	[129]
PP 6315, PP 6312, PP 6310,	MFR 1500, MFR 1200, MFR 1000	M-ESP	–	Top electrical heating ring 230 °C bottom heating ring 210–250 °C	60 (Collector + ve)	4.47 to 41.72	[130]
Isotactic PP Atactic PP	Mw 12,000 Mw 19,600	M-ESP	–	Extruder; 200 °C	6–15/cm (Collector + ve)	3.55–13.06	[131]
PP	MFR 2000	M-ESP	–	Electrical heater/feed inlet 180 & 200, nozzle 230 °C	27, 30, 33, and 36 (Electrode + ve), 65 (Collector -ve)	3.35 to 7.14	[118]
PP	MFR 2000	M-ESP	–	Heating coil/nozzle 240 °C	20–50 (Electrode + ve)	3 to 6	[132]
PP	MFR 1100	M-ESP	–	Electric heater/metal hopper, nozzle 185–260 °C	17–29 (Nozzle tip + ve)	2.73 to 6.00	[133]
Isotactic PP	MFR 3.1	M-ESP	Na, Ca, Mg and Zn stearate	Extruder 180 & 200 °C	135 (Electrode + ve)	1.2 to 3	[134]
PP	MFR 2000	M-ESP	Arstar, nano-CaCo_3_, SDBS	230 °C	30 (Top plate), 60 (Bottom plate)	1–8	[135]
PP	Mw 195,100 MFR 35	M-ESP	–	Electric heating/syringe 230, needle 280–290 °C	10–20 (Needle + ve)	< 1	[136]
PP	MFR 1000 (PP)	M-ESP	SO, PEG, PDMS	Metal barrel electrically heated; 200 °C	48 (Collector − ve)	0.88 to 5.25	[137]
Isotactic PP	Mw 210,000 MFR 15	M-ESP	Irgatec	Glass syringe/heat gun; 270–320 °C	20 (Spinneret − ve)	0.84–8.6	[102]
PP	Mw 100,000 MFR 2000	M-ESP	–	Electromagnetic heater/extruder 200–240 °C	25–45 (Collector -ve)	0.79	[101]
Isotactic PP	–	M-ESP	TiO_2,_ Na-stearate	Electrically heated/glass syringe 210–305 °C	25–30	0.7–0.8	[138]
PP	MFR 35 (EVOH)	M-ESP	EVOH	CO_2_ laser	35 (Electrode + ve)	0.66	[139]
Isotactic PP	MFR 12, 35 and 75	M-ESP	EVOH	CO_2_ laser	20–70 (Electrode + ve)	0.64 to 4.46	[104]
PP	Mw 80,000 MFR 1500	Electrostatic-melt blown	–	Electric heating/extruder 265 °C	0–40 (Collector -ve)	0.60	[90]
PP	MFR 1200 MFR 1800	M-ESP	DTAB	Cartridge heater/melt reservoir 320–360 °C	50–95 (Collector)	0.4 to 14	[140]
PP	MFR 25	M-ESP		Spinning head 270–300 °C	30–40	0.35 to 5.96	[141]
PP	Mw 55,509 MFR 2000	M-ESP	SO and NaCl	Metal barrel electrically heated; 200 °C	48 (Collector − ve)	0.31 to 4	[14]
PP	Mw 190,000 MFR 35	M-ESP		Radiant heater; 250–300 °C	100–3000 kV/m (Collector ± ve, spinneret ± ve)	0.3 to 30	[100]
PP homopolymer	MFR 1200	M-ESP	Low modulus PP, Irgastat, Sodium stearate	Heating chamber/syringe 210 °C	35–55 (Collector -ve)	0.21	[103]
Isotactic PP	MFR 35	M-ESP	PVB	CO_2_ laser	40–45 (Electrode + ve)	0.181	[113]

PP; polypropylene, Mw; molecular weight, MFR; melt flow rate (at 230 °), MVR; melt volume rate (at 230 °C), S-ESP; solution electrospinning, M-ESP; melt electrospinning, o-DCB; o-Dichlorobenzene, Bu4NclO4; tetra-n-butylammonium perchlorate, DTAB; Dodecyl trimethyl ammonium bromide, SO; Sodium oleate, SDBS; Sodium dodecyl benzene sulfonate, PEG; Poly (ethylene glycol), PDMS; Poly (dimethyl siloxane)

Recently, Daenicke et al. [103] combined all possible ways for the reduction of melt electrospun fiber diameter and reported ultra-fine PP nanofiber with only 210 nm in diameter. They accumulated the low molar mass polyolefin “L-Modu” as a viscosity reducer, “Sodium stearate” as a polarity improver, and “Irgastat P16” as an antistatic agent being added to PP with a high MFR (1200 g/10 min) and conducted the electrospinning process under a controlled environment between room temperature and 120 °C. Using electrostatic spinning the lowest diameter (181 nm) polyolefin (PP) nanofibers to date has been reported from our lab [113] where PVB was employed as a spinning aid to improve the electrical conductivity of polymer melt.

### Polyolefin blends nanofiber

Polymer blend generally adds synergistic, expedient, and often impossible functionalities to the base material that merge with easy processing, commercial viability, and applicability [22]. Blending with proper material is the most effective technique to curtail the hurdle of electrostatic spinning of polyolefins considering the non-polarity, high melt viscosity, and low productivity. Table 3 demonstrates an up-to-date shortlist of electrospun fiber preparation from different polyolefins blend. M-ESP method along with PP as a base material is mainly used in the case of polyolefin blend fibers preparation. SEM images of melt electrospun PP/PVB and PE/PVB blend nanofibers are shown in the Fig. 7e, f, k. The blend components are generally mixed with polyolefins prior to the spinning using a melt mixer at a higher temperature over the melting point of polymers and are delivered to the electrospinning device as film [104], rod [139], or particle [142]. The existence of blend component in the electrospun fiber ensures diversified applications and the removal after spinning results in a drastic drop of polyolefins fiber diameter with an increased specific surface area. Cao et al. [116] manufactured melt-electrospun microfiber webs using styrene-acrylonitrile (SAN) copolymer and PP blends and suggested then as a perfect material for protective clothing, filtration media, and composite reinforcement. A novel conductive fibrous membrane has been prepared by blending multiwalled carbon nanotube with PP that exhibits improved fiber spinnability, tensile strength, tensile modulus, electric conductivity, dielectric constant, and hydrophobicity [142]. In addition, using clay particles and nano-TiO_2,_ an antibacterial polypropylene filter medium has been made and the suitability of the filter media is also confirmed for the elimination of pathogenic microorganisms [42]. Fujii et al. [104] prepared PP/EVOH/PP blend fiber from the laminated polymer films that transformed into a PP hollow tube with an increased surface area after EVOH removal. Recently, PP/PVB and LDPE/EVOH blend fibers have been prepared using laser M-ESP technique with high productivity and then obtained PP and LDPE nanofibers with a record low diameter by removing blend components from the as-spun fibers [110, 113].

**Table 3:** Electrospinning of polyolefin blend with processing technique and resultant fiber diameters

Material used	Material property (Mw in g/mol, MFR in g/10 min	Processing technique	Heating temperature	High voltage supply (kV)	Fiber diameter (µm)	References
PP/PVB	MFR 16/Mw 200,000	M-ESP	Heating syringe 330 °C	35 (Collector)	12 to 18.4	[143]
LDPE/EVOH	MFR 13, 23, 45, 70, 145 (LDPE) MFR 14 (EVOH)	M-ESP	CO_2_ laser	40 (Electrode + ve)	6.3 to 11.4	[110]
PP/Styrene– Acrylonitrile	Mw 7000–8000, MFR 1500/ MFR 2.2	M-ESP	Top electrical heating ring 230 °C bottom heating ring 210–250 °C	60 (Collector + ve)	5 to 10	[116]
LDPE/PVB	MFR 13 (LDPE)	M-ESP	CO_2_ laser	40 (Electrode + ve)	2.89 to 7.85	[109]
PP/Arstar, PP/nan-CaCo_3_, PP/SDBS	MFR 2000 (PP)	M-ESP	230 °C	30 (Top plate), 60 (Bottom plate)	1 to 8	[135]
Isotactic PP/CNT/PL	MFR 15 (PP)	M-ESP	Electrical heating ring/cylinder 265 °C	60 (Collector + ve)	1 to 3	[142]
PP/EVOH	MFR 35 (EVOH)	M-ESP	CO_2_ laser	35 (Electrode + ve)	0.98 to 1.66	[139]
PP/PVB	MFR 35 (PP)	M-ESP	CO_2_ laser	40 (Electrode + ve)	0.95 to 1.4	[113]
PP/EVOH/PP	MFR 12, 35, 75 (PP) MFR 14 (EVOH)	M-ESP	CO_2_ laser	20 -70 (Electrode + ve)	0.64 to 1.08	[104]
PP/Clay, PP/TiO2	MFR 25 (PP)	M-ESP	Cartridge heater/melt reservoir at above PP melting point	20 (Collector)	0.242 to 0.31	[42]

SDBS; Sodium dodecyl benzene sulfonate, PL; Paraffin liquid

### Miscellaneous polyolefin nanofibers

Polyisobutylene (PIB), a member of polyolefin family, is a versatile synthetic elastomeric macromolecule. PIB nanofibers are highly desirable due to their hydrophobicity, non-cytotoxicity, and resistance to biofilm formation and fibroblast cell adhesion. The aforementioned characteristics make their nanofibrous mats viable prospects for application in the field of biomedical engineering [144]. A linear triblock copolymer of PIB called L_SIBS composed of poly(styrene-b-isobutylene-b-styrene), is widely employed as a drug-releasing coating on the TAXUS™ coronary stent [145]. The Puskas group developed other generations of thermoplastic elastomer (TPE) following the success of L_SIBS. These include Arbomatrix©, which consists of a branched (arborescent or dendritic) PIB core and end blocks made of polystyrene or its derivatives [146, 147], and Allomatrix©, which is composed of poly(alloocimene-b-isobutylene-b-alloocimene) [148–150]. However, these TPEs are not available for commercial purchase. The drug-filled nanofibrous mat is widely recognized for its ability to accelerate the release rate due to its enhanced surface-to-volume ratio. Nevertheless, Liu et al. reported that pure L_SIBS could not be electrospun, due to its non-conductivity [151, 152]. Subsequently, Lim et al. demonstrated the successful electrospinning of L_SIBS in combination with Arbomatrix. This rubbery fiberous mat has 2.7 MPa tensile strength at 537% elongation, is hydrophobic, and also cytocompatible in cell culture [153]. Following that, a novel approach has been devised to generate self-supporting fiber mats through the electrospinning process utilizing low-molecular-weight polyethylene glycol (PEG) in combination with Arbomatrix© and Allomatrix© [154, 155]. Moreover, the ZAF-loaded Arbomatrix©/PEG microfiber, which has a diameter of 4.197 ± 0.580 µm, was produced via electrospinning [154]. The electrospinning of Allomatrix© is considerably simpler than Arbomatrix© [148, 149, 156]. Arbomatrix© and Allomatrix© possess a significantly greater molecular weight compared to L_SIBS and have the ability to be strengthened with fillers [157]. Commercially, only L_SIBS is available. Dóra Barczikai and colleagues electrospun a novel self-supporting mats of 203.75 and 295.5 g/m^2^ that combines L_SIBS with zinc oxide (ZnO), using butyl rubber as a filler for reinforcement [144].

Poly(4-methyl-1-pentene) (PMP) is a polyolefin that exists in multiple forms and is characterized by being non-toxic, non-contaminating, and lighter than water. It exhibits a high level of transparency to light, exceptional electrical dielectric features, powerful resistance to chemicals, high tensile strength, and a high degree of gas permeability [158]. These characteristics and its higher melting temperature, bestow it advantage over other polyolefins and make a potential candidate for labware applications. However, due to its insolubility in organic solvents, high chemical resistance, and electrical resistivity, electrospinning PMP into nanofibers remains tough [159]. Lee et. al. prepared PMP nanofibrous membrane using four different solvent systems: cyclohexane, cyclohexane/acetone mixture, cyclohexane/dimethyl formamide (DMF) mixture and cyclohexane/acetone/DMF mixture have been investigated [160]. They reported the strong dependency of solvent type and amount on the shape and morphology of nanofiber. Electrospun poly(1-butene)/PMP blend nanofiber has also been prepared with a twisted-ribbon structure having irregular twisting points along the length of the fibers [161]. Nevertheless, the production of PMP nanofibers without any beads remains to be a challenging endeavor. According to Wahab Jatoi's research, PMP high strength and bead-free nanofibers have been produced using the electrospinning technology, making them suitable for technical and industrial use [159]. Recently, Narejo et al. [162] developed smooth and bead-free co-electrospun cellulose/PMP nanofibers membrane followed by deacetylation with improved mechanical strength.

### Potential challenges and remedies

**Fig. 8:**
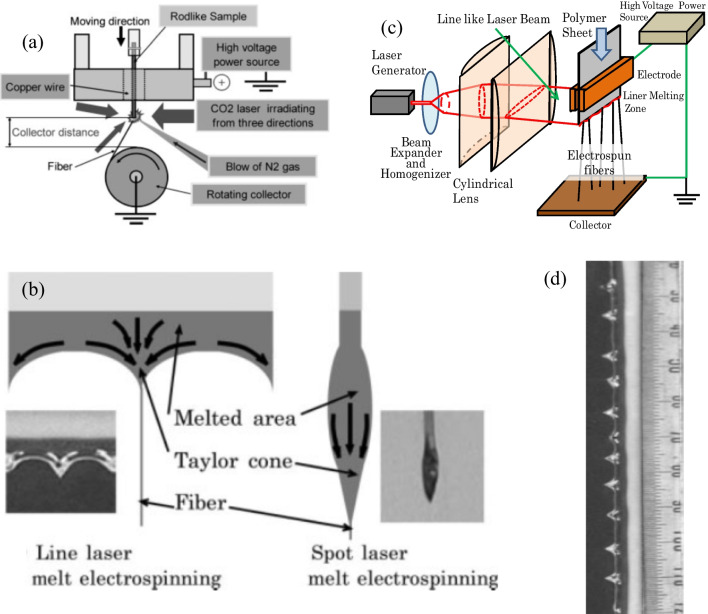
Development of laser M-ESP system; **a** M-ESP system with a spot CO_2_ laser melting device [166]. **b** Flow and Taylor cone formation of molten polymer in the laser melt-electrospinning process [168]. **c** M-ESP system with a line-like CO_2_ laser melting device. **d** Multiple Taylor cone formation for line laser [104]

Nanofiber with as low as diameter results in an extremely high specific surface area [13], ensuring good barrier characteristics against microorganisms and fine particles, with smooth and excellent covering. These exclusive characteristics make polymer nanofibers potential candidates for current applications in various interdisciplinary domains. The fabrication of polyolefin nanofibers with a diameter smaller than 500 nm poses an immense challenge due to the lack of appropriate solvents for solution electrospinning and the high melt viscosity encountered in melt-electrospinning. As it relates to developing polymeric fiber in nanoscale dimension, the solution electrospinning technique is superior since it is efficient, simple, and flexible. However, the solubility of polyolefin is limited to a small number of very hazardous solvents, which significantly impacts its suitability for large-scale production and limits its potential applications in several sectors particularly in the field of biomedical engineering [19, 163, 164]. The utilization of M-ESP addresses the concerns associated with solvents, rendering it a more environmentally sustainable, adaptable, and cost-effective approach to produce polyolefin nanofiber [102, 131, 165]. Nevertheless, the main hurdles in achieving polyolefin nanofibers in M-ESP arise from the high viscosity, low conductivity, and exit swelling of polymer melts [77]. Numerous endeavors have been undertaken by researchers to surmount these challenges, with a particular focus on process refinement and the incorporation of appropriate ingredients. Ogata and colleagues have effectively devised a novel variant of the M-ESP approach by employing a CO_2_ laser melting apparatus, [166] system is shown in the Fig. 8. This technology facilitates a nozzle- and solvent-free electrospinning system, resulting in reduced energy consumption and the elimination of the requirement for molten polymer preparation in a reservoir [95]. Its instant, highly controlled, and localized laser heating system avoids the thermal degradation of polymer melt by long term heating [167]. Shimada et al. updated the feed system with sheet-like polymer and laser shape from spot to linear to make multiple Taylor cones and drastically enhanced electrospun nanofiber productivity [168].

The diameter of melt-electrospun polyolefin nanofibers has been slightly reduced by the process modifications alone, however, there are still concerns regarding the fiber diameter. In this regard, the incorporation of viscosity-reducing additives such as “Irgatec” [102] and “L-Modu” [103], polarity improvers like “Sodium stearate” [103], and spinning aids like “EVOH” [109, 110] and “PVB” [113], in conjunction with polyolefin, has demonstrated significant prospects for reducing fiber diameter. The incorporation of spinning aids, which were subsequently eliminated after electrospinning, has been established as more efficient and environmentally friendly approach. This technique has produced the smallest recorded PE and PP nanofibers, with diameters of 191 and 181 nm only, as shown in Table 1 and 2.

## Applications of polyolefin nanofiber

Polyolefin nanofibers are a popular material due to their numerous advantages, including chemical resistance, low weight, and affordability. This versatile material has a wide range of potential applications, including filtration, oil–water separation, bio-medical engineering, protective clothing, and battery separators, to name a few. Among the various types of polyolefin nanofibers, modified PP nanofibers are particularly highly consumed in the market [169]. Polyolefin nanofibers are known for their exceptional mechanical and thermal properties, making them ideal for a variety of industrial uses and a popular choice for scientists and engineers who are working on developing novel materials for innovative applications.

### Filtration and separation

A safe environment and sound health are highly appraising to the modern civilization where water and air pollution are alarming issues. The surface water can be polluted seriously by oil, waste materials, nuclear wastes, wine, beer, bacteria, algae, protozoans, etc. [170–172]. Filtration is the most effective, easiest, and widely used technique, especially for water and air purification. Fibrous filter media is more advantageous for high-performance filtration that may be woven or nonwoven types [173, 174]. Nonwoven filter is easily prepared by randomly aligned fibers ensuring high filtration and separation performance [175]. The filtration efficiency is indirectly related to the fiber diameter where the finer fiber is more potential for smaller mean pore size as well as acute separation [176, 177]. Consequently, nanofiber plays a great role over microfiber filters because of some important parameters such as high specific surface area, smaller mean pore dia, lower drag force, and high permeation flux [178, 179]. Moreover, the nanofibrous membrane also exhibits the unique interconnectivity of pores and effectively rejects contaminants even in nanoscale dimensions. Consequently, the particles smaller than 100 nm can be separated from air and water using a nanofibrous membrane [17, 76]. The SEM image of the nanofibrous membrane is shown in Fig. 8a, b. Among the polyolefins, PP micro and nanofibers are extensively studied to prepare high-performance membranes for liquid filtration. Li et al. [132] investigated the effect of fiber orientation on the water filtration properties of the PP fibrous membrane. The oriented melt electrospun PP fibers resulted in a smaller average pore diameter showing a higher rejection percentage with a better permeates flux. Uppal et al. [76] prepared a PP nanofibrous membrane through the modular melt-blowing technique. They observed the improved filtration efficiency of the membrane for finer fiber diameter due to the greater specific surface area and the smaller pore size irrespective of the sample's basic weight. In addition, Zhu et al. [78] observed the outstanding separation performance of TiO_2_ and dyeing wastewater with the excellent pure water flux of PP micro/nanofibrous membrane over the microporous membrane. Recently, Zakaria et al. [114] developed a membrane from superfine PP nanofibers that is highly desired for efficient filtration of industrial wastewater. In that study, a PP nanofibrous membrane with an average fiber diameter of 228 nm was used, and it was shown that the fiber diameter has a direct effect on its properties and how well it cleans textile wastewater. Compared to a commercial PP fibrous membrane, their developed PP nanofibrous membrane turned out to be more promising. In a different study, melt-blown activated PP nanofiber was found as an effective adsorbent for the removal of 2,6-dichlorophenol (a toxic and corrosive wastewater pollutant) because of its high surface-to-volume ratio, better porosity, and good mechanical properties. The Langmuir model was found to be the best fit for the adsorption isotherm with a maximum adsorption capacity of 44.44 mg/g. Adsorption increased the PP nanofiber diameter and added phenolic chemicals to the fiber surface [180].

Emission of industrial oily wastewater and marine oil spillage accidents are not only serious threats to the environment but also a waste of precious resources [181–184]. However, the conventional oil/water separation materials including coated-mesh [185], fabrics [186], and sponge [187, 188], are limited to large pore size, low separation efficiency, flexibility, and energy consumption. The large scale availability even low cost and the inherent hydrophobicity, yet oil-friendliness of PP renders it as the most suitable marine oil-spill cleaning material [189–192]. Recently, PP-based membranes have been potentially employed for oil-spill cleanup and their superhydrophobic performance over the traditional polymers is valued enormously [193, 194]. Li et al. [118] prepared fibrous membranes using electrospun ultrafine PP fibers and reported approximately six to seven times higher oil-sorption capacities over commercial melt-blown PP nonwoven mat for motor and peanut oil cleanup. Additionally, they found above 97% oil recovery even after seven sorption/desorption cycles with a superior mechanical performance that ensures its high reusability and recoverability. Anyang et al. [129] also revealed a highly reusable (around 30 cycles) cuprous oxide/PP fibrous membrane that exhibited a remarkable oil/water separation efficiency above 94%. Figure 8c presents their superhydrophobic (WCA = 151.8 ± 2.1º) fibrous membrane with the simulation of the oil/water separation process. In a recent study, Semilin et al. [195] have showed how PP micro/nanofiber (PP-MNF) can be used to get oil out from the wastewater of a palm oil mill. It was demonstrated that PP-MNF has a high adsorption capacity for residual oil, and the oil was successfully recovered by hand pressing and solvent or supercritical CO_2_ extraction techniques. The recovered oil had a similar quality to crude oil and was free of contamination from polypropylene. After extraction, PP-MNF remained unchanged physically, suggesting its commercial use in residual oil recovery from palm oil mills.

**Fig. 9:**
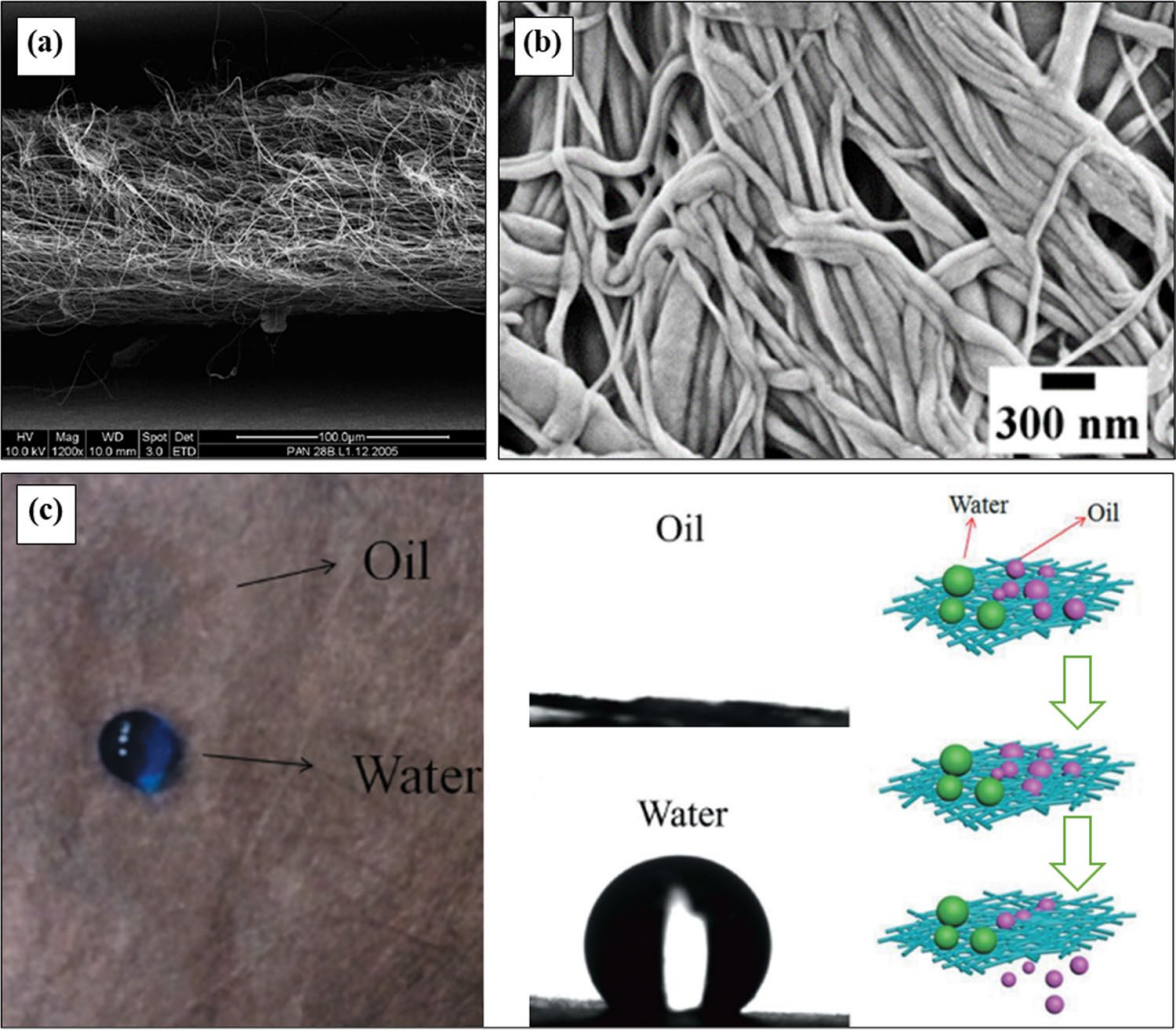
SEM image of (**a**) nanofibrous membrane [17], **b** PP nanofibrous membrane with higher magnification [114], **c** water and oil droplets on the surface of Cu_2_O/PP fibrous membrane and the demonstration of separation process [129]

The global demand for uranium is increasing aggressively in the nuclear fuel industry and nuclear weapons construction day by day [196]. That generates huge contamination of uranium ions in the seawater resulting in a dangerous toxic and radiological impact for all living organisms, including human beings throughout the planet. So the removal of uranium from seawater is a crucial assertion for a sustainable environment [197–199]. Among various methods, the adsorption process is more cost-effective and highly efficient for uranium removal from seawater [200]. PP is a highly chemically resistive polymer having good mechanical strength, which is more prominent for uranium adsorption but its non-polarity is a major drawback. For this reason, it is necessary to graft the functional groups on their surface with a high percentage [201]. The grafted PP nanofiber surface was produced by Ashrafia et al. [82] using the high-energy electron beams pre-irradiation process with acrylonitrile and methacrylic acid monomers. The modified PP nanofiber is seen as a promising and effective adsorbent for removing uranium ions from seawater and other aqueous solutions. Its large surface area and high percentage of functional monomer grafting make it highly efficient in this process (Fig. 9).

**Fig. 10:**
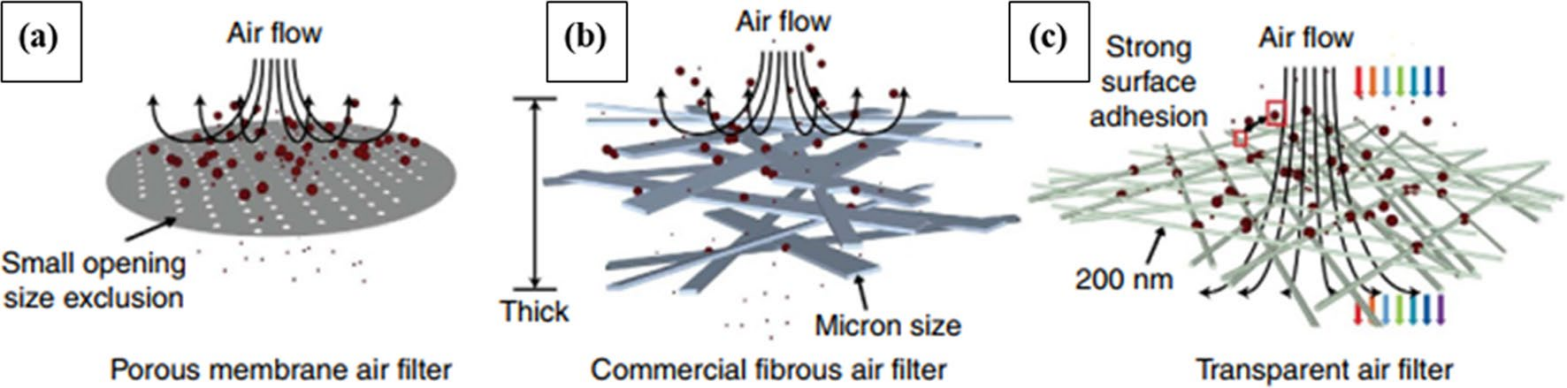
**a** Schematics of porous membrane air filter. **b** Schematics of bulky fibrous air filter capturing PM particles by thick physical barrier and adhesion. **c** Schematics of transparent air filters that capture PM particles by strong surface adhesion and allowing a high light and air penetration [204]

Both outdoor and indoor air pollution by a complex mixture of extremely small particles and liquid droplets is a serious concern for human bronchi and lungs [202]. PP, carbon, and copper are all widely used materials in commercial air filters. Usually, two types of air filters namely porous membrane (Fig. 10a) and fibrous nonwoven mesh with many layers of thick fibers ( Fig. 10b) are commonly used, however, most of those are unsuitable due to their less porosity and poor air permeability [203]. Liu et al., introduce a novel transparent fibrous air filter by depositing electrospun nanofibers on a glass wire mesh for indoor air protection, shown in Fig. 10c. The main function of this technology is high filtering efficiency, good optical transparency, low resistance to airflow, and lightweight than the existing fibrous filters [204]. However, they found poor particle-capturing capability for the PP transparent filter due to its coarser fiber diameter. Instead, Pu et al. [90] developed PP fiber with an average diameter of 600 nm and prepared fibrous filter media with a smaller mean pore size. They observed the improved filtration efficiency and suggested it as a potential air filter.

### Biomedical engineering

Since viruses and other biomolecules are in nanometer dimension the materials in nanoscales are highly desired to be well explored in biomedical engineering field [205]. In the organic thermoplastic polymer region, the polyolefin is traced as the largest class in terms of diversities in application [206]. They are considered commodity polymers because of their large volume production and based on the consumption in the globe everyday [207]. In the medical area, the application of polymeric materials has drastically replaced the portion of conventional materials because of the superiority in properties, ease of production, availability, and scope of manipulation in properties [208]. From heart components to liver parts, dentures to hip, tracheal to facial prostheses, and knee joints the application has become vast [209]. But it is a necessary consideration that before using a material in biomedical engineering they must possess certain qualities ideal for using in those techniques [208, 210].

**Fig. 11:**
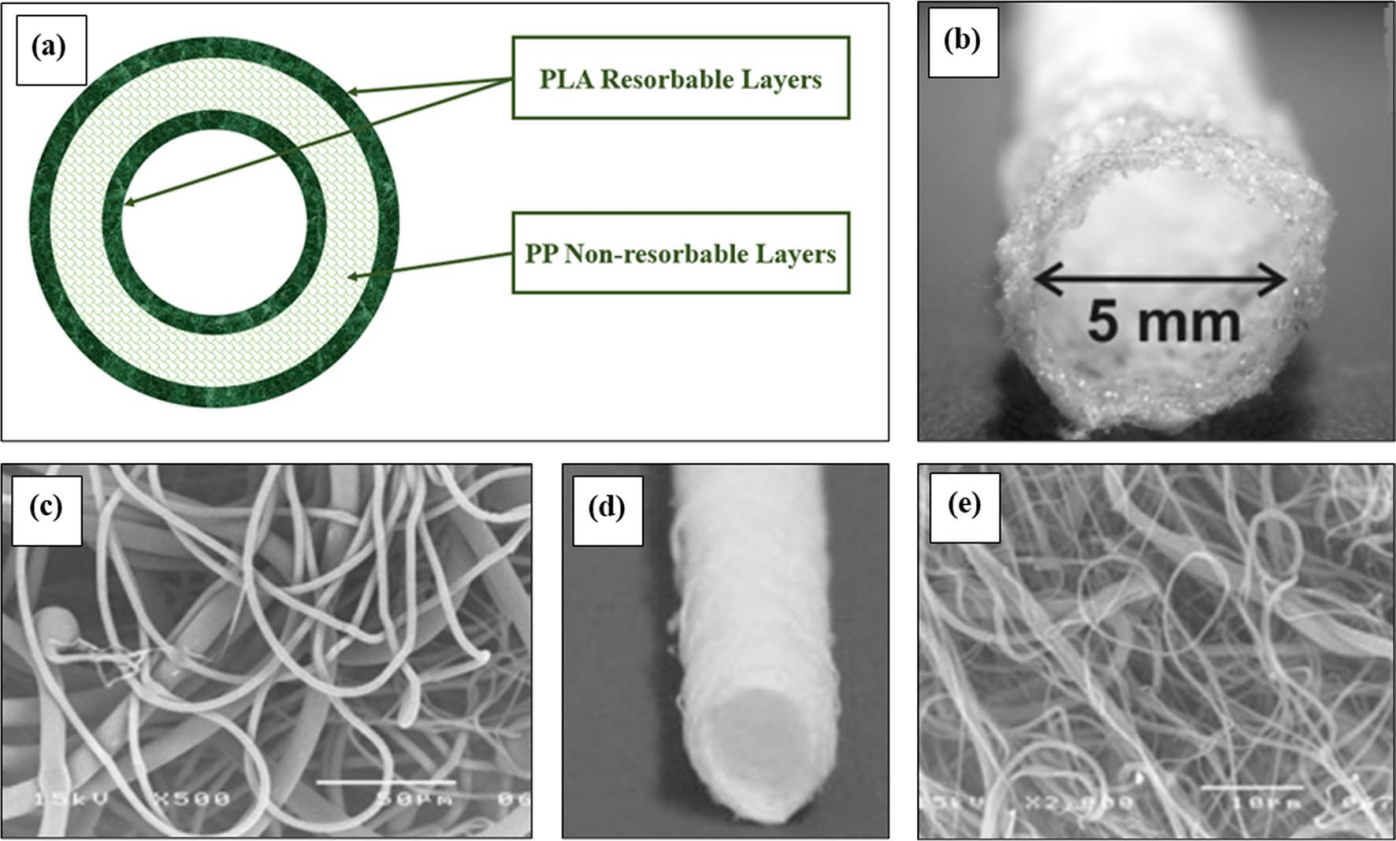
**a** Concept of Vascular Prosthetics, **b** macroscopic view of tubular model made of the Moplen 462R PP, **c** SEM image of external surface of tubular model made of the Moplen 462R PP, [128]. **d** PP tubular structures prepared by melt-electroblowing technique, **e** SEM image of external surface of tubular model prepared from melt-electroblown PP nanofiber [141], Curtesy of Wiley Periodicals, Inc

In the whole world cardiovascular diseases have become one of the significant causes of death often resulting from arteriosclerosis. American Heart Association has shown these diseases as one of the biggest reasons for mortality [211]. Several researches have been conducted regarding the development of vascular prosthetics using polyolefin materials. A group of scientists felt the necessity of an artificial vessel supply in the place of defective arteries and they have developed a model of cardiovascular graft [212]. They used a PP nanofiber-based base structure in the middle layer and polylactic acid (PLA) as a sophisticated outer and inner layer. The model has been demonstrated in Fig. 11. The PP inner layer is stated to be the non-resorbable middle layer to hold the structural identity of the model. The application has been proposed for securing heart disease and bypass surgeries.

The application of polyethylene terephthalate and expanded polytetrafluoroethylene are well-known [213]. But when the vascular vessel diameter goes small and less than 6 nm the rate of success in different surgical reconstructions gets challenging [214]. Mazalevska et al. [128] have applied a polypropylene-based flat structure to design a model of a vascular graft in melt electrospinning technique. The method for melt electrospinning comes for property manipulation and ease of reducing the harmful effects of solvent.

A different study on the application of polypropylene-integrated vascular graft materials conducted by Chrzanowska et al. [141] has shown how tubular structures of small diameter in cardiovascular treatment can be developed using PP and PLA. Three techniques were implied in the study: melt blowing, melt-electro blowing, and melt electrospinning in the synthesis of the tubular structure. They have shown that the shortest diameter of the fibers for the PLA and PP tubular structure was obtained from the first method, melt-blown structure. The PP tubular structure diameter was approximately 0.38 ± 0.19 μm. They also tested the biocompatibility of the materials to explain the impact of biological decomposition on the degradation of the prostheses. It has been found that PIB-based elastomeric nanofibrous mats with outstanding hydrophobic characteristics have superior tissue and blood compatibility, making them the most suitable for biomedical applications [153, 215]. Copolymers of PIB, including L_SIBS, Arbomatrix©, and Allomatrix©, are well-known for their use as nanocoating materials that can hold different drugs in coronary stents. Yet their slow drug release rate makes them less effective [216]. Later on, the PIB nanofibrous mat containing drugs emerged as a promising solution to address this problem. Zafirlukast (ZAF), a model drug, has effectively encapsulated within Arbomatrix© fiber mats, exhibiting a release rate above 90% [154]. Lim et al. electrospun a hydrophobic and cytocompatible nanofibrous mat combining with both L_SIBS and Arbomatrix demonstrated tremendous potential for drug delivery and tissue engineering scaffolds [153]. Dóra Barczikai and coworkers developed a new COVID-19 mask that is non-cytotoxic, highly breathable, and made by combining L_SIBS, zinc oxide (ZnO), and butyl rubber using electrospinning [144].

Nano-scaled polyolefin component also has a vast range of application for the antimicrobial or antibacterial functionalities obtained by various synthesis and processing mechanisms. Shafiee et al. [42] have developed clay particles and nano TiO_2_ containing nano polypropylene filtration media. nTiO_2_ (TiO_2_ nanoparticles) have both a high fraction of surface atoms and a high specific surface area. Their unique properties like dielectric, optical, photocatalytic properties, and physiochemical features result in their use in the separation of various harmful organic materials and bacteria from water and air. On the other hand, clay particles are also highly used in different area from hygiene to industrial consumables and health safety cares [217]. The clay imparts high surface area, good cation exchange capacity, colloidal properties, and the affinity for absorbing both inorganic and organic substances. The study by Shafiee et al. [42] has examined the antibacterial properties of both TiO2 and clay particles containing nanopropylene with *E. coli* bacteria and observed preferential performance with clay particles.

Silver is a prominent material in the application of antibacterial functionalities. Also, it has been proven that silver can kill more than 650 disease-related organisms in the human body. They are known as one of the safer antimicrobial agents compared to many organic compounds [218, 219]. The smaller silver particles in the nanoscale exhibit more surface area and specific functionalities with the decreasing particle size [220, 221]. Yeo et al. [222] have developed some nanocomposite fibers with inorganic and organic materials to judge their antimicrobial properties. They developed sheath-core type nanocomposites with PP masterbatches and PP chips having variations in silver nanoparticle concentration. They have found better antibacterial performance for those sheath-core structures where the PP/Ag masterbatches are used in the sheath rather than the core of the structures. Better exposure on the outside resulted in better antibacterial activity.

### Protective clothing

Protective clothing is an essential apron for agricultural laborers, industry employees, military personnel, law enforcement officials and hospital nurses who work with pesticides, poisonous chemicals, and hazardous substances [223]. Polypropylene-based protective garments, such as nonwoven, knitted, woven, microporous, and monolithic materials, are commonly used to decrease employee skin exposure to pesticides due to their low surface energy, chemical inertness, lightweight, and low cost [224]. The efficiency of protective materials is determined by their barrier performance, which has a negative connection with air permeability and thermal comfort [225]. Barrier performance is poor in nonwovens with high air permeability, whereas microporous materials and tightly interlaced woven offer superior barrier performance but lower air permeability [226]. Protective garment materials should therefore have reliable barrier performance with breathing comfort [224]. Schreuder-Gibson et al. [227] demonstrated enhanced aerosol protection via a fine layer of electrospun fibers, focusing on barrier materials to penetrate chemical warfare agents in aerosol form. The produced webs provided good aerosol protection without a significant change in the moisture vapor transport system.

**Fig. 12:**
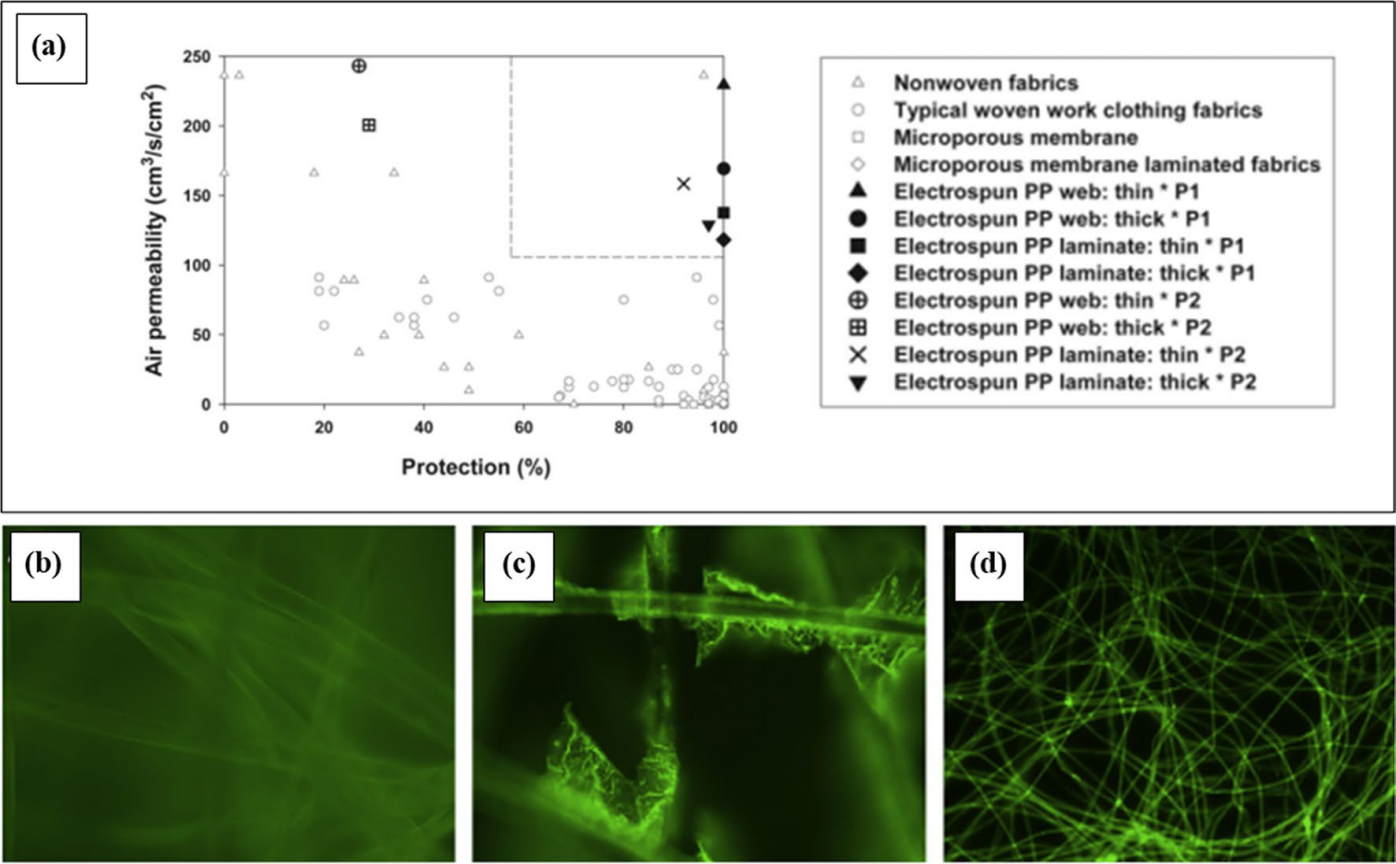
**a** Air permeability and protection performance of electrospun polypropylene web/laminates compared with existing PPE materials (PP = polypropylene; P1 = pesticide mixture 1; P2 = pesticide mixture 2, protection % = 100—penetration %) [136], **b** protein adsorption results of PP non-woven webs, **c** melt-electrospun fiber webs, and (**d**)) solution-electrospun fiber webs using bovine serum albumin labeled with fluorescein isothiocyanate (BSA-FITC) [119]

Lee et al. [136] developed another protective textile material with good barrier performance and thermal comfort qualities for agricultural workers. When compared to currently available personal protective equipment (PPE), electrospun PP webs and laminates displayed a combination of superior protection and good air/vapor transmission properties as shown in Fig. 12a. Liquid protection is another essential aspect of barrier clothing for everyone, not just employees. Jinhao et al. [143] fabricated a novel melted polyvinyl butyral (PVB) bonded PP composite fibrous mat, where PVB and PP were mixed at different percentages to increase the structural stability and mechanical properties of a pristine PP fibrous mat system. The mechanical properties, including tensile stress, and tearing strength of composite fibrous mats were improved significantly due to the bonding between PVB and PP molecules in the fiber. The fabricated composite material has satisfactory breathability with sufficient resistance to water penetration because the designed composites exhibited better hydrophobicity than pure PP fibrous mats. Cho and co-workers [119] introduced an isotactic polypropylene fiber applied both solution and melt electrospinning using an elevated temperature setup. Their electrospun PP webs exhibited super hydrophobicity with a better water contact angle, which was substantially higher than those of a commercial PP nonwoven web. They also observed the enhanced Bovine serum albumin (BSA) protein adsorption by electrospun PP fibers that indicated its promising anti-bacterial activity shown in Fig. 12b, c, d. For this reason, this has been suggested that the electrospun PP nanofibrous webs can offer excellent protective performance without losing permeability [119]. For skin contact products, PP fabrics must be soft, but tailoring this softness is difficult. Zhen et al. [228] examined the effects of adding PE to PP during melt-blowing on the morphology and properties of the resulting micro-nanofibrous fabrics. They showed that the inclusion of PE altered the crystalline integrity of PP and produced a micro-nanofiber structure with diameters between 1.3 and 3.2 μm. It has been recommended that their micro-nanofiber fabric be used in skin-contact products like baby diapers and personal hygiene items.

### Battery separators

**Fig. 13:**
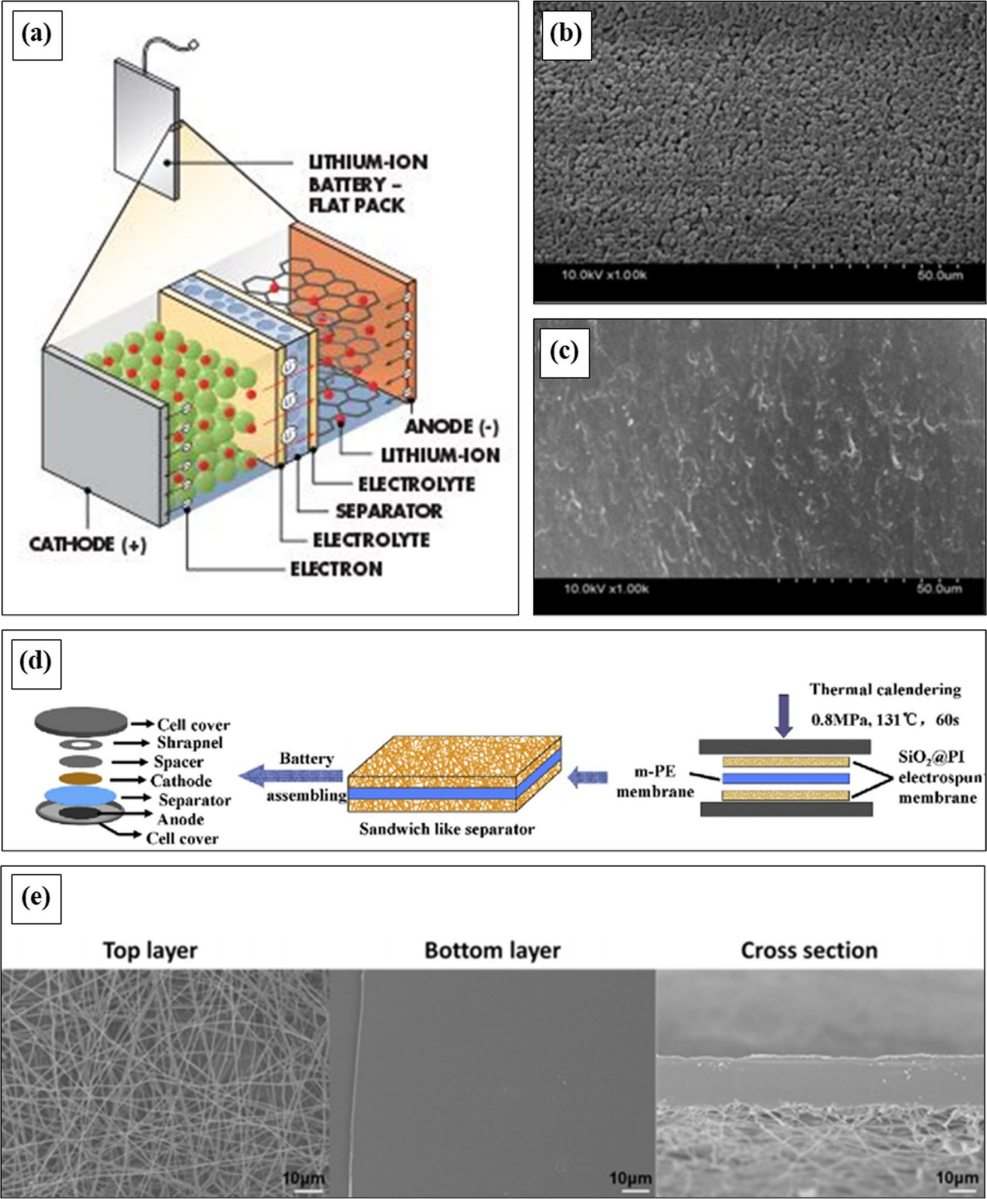
**a** Different parts of a lithium-ion battery, SEM image of PE membrane, **b** before and (**c**) after heat treatment [231], **d** schematic view of the tri-layered composite separator and battery assembling [233], **e** FE-SEM image for PET/PP-22% after shutdown [234]

Battery separators are a kind of polymeric membrane that is placed between the negatively charged cathode and the positively charged anode to prevent electrical short-circuiting. Separators are not electrically conductive, but they allow ions to pass through freely, and therefore, always act as isolators. Nowadays, separators are essentially being integrated with modern rechargeable battery, and act as a barrier between anode and cathode. A lithium-ion battery (LiB) belongs to the family of rechargeable battery types, where lithium ions are employed as a key component of its electrochemistry. Currently, LiB has received much more attention in the energy source market due to its high durability, larger power density, and good electrochemical properties [229]. This type of battery is commonly used to operate various portable devices like electrically powered cars, computers, phones, and other electric gadgets [230–233]. Different parts of a LiB are demonstrated in Fig. 13a.

In the LiB system, the microporous separator film or sheet is interposed between anode and cathode that is filled with an electrolyte of conductive liquid [235, 236]. Pore size, permeability, shutdown capability, retention and absorption of electrolyte, chemical, and mechanical stability have been stated as the major performance indicators of a battery separator [237, 238]. Hence, the selection of an efficient separator is considered a crucial issue for the safety and restoration of high energy of LiB system [239, 240]. Accordingly, over the decade polyolefin-based membranes or nonwovens have become the most dominant battery separator because of their good mechanical activity and chemical stability [231, 241]. To prepare such types of nanofibrous membranes or nonwoven separators electrospinning is the easiest and straightforward technique. In short, the nonwoven type battery shutdown layer is expected to be highly demandable for LiB assistance [242–244].

Currently, PP and PE nanofibrous mats, as shutdown layers or separators are considered a perfect battery stuff [245], however, the electrospinning of these polymers is highly complicated due to the poor solubility in common solvents. In this regard, poly-1-butene (PB) is more beneficial for use as a shutdown layer of LiB because of its better electrochemical properties and a similar melting point of PP and PE [161, 246]. Moreover, it has better solubility among olefin family members ensuring the easy electrospinning [247, 248]. Jeong et al. [230] prepared an electrospun fibrous mat using homo and copolymer of poly-1-butene (PB) on a polyethylene terephthalate (PET) support and proposed as a potential battery separator. By adjusting the monomer ratio in the copolymer of PB the melting point can be altered. Hence, they found the copolymer-based PB nonwoven mat more advantageous for its customizable shutdown capability.

Polyolefin battery separators are extensively used because of their high chemical resistance and super mechanical performance. However, the poor electrolyte wettability and easy melting at elevated temperatures are the major drawbacks of pure polyolefin as separators, occasionally causing an internal short-circuit [249–254]. To resolve the issue, Cai et al. [234] prepared a composite (PET/PP) separator, which exhibits better electrolyte affinity and mechanical stability. They have prepared separators with a different ratio of PET electrospun nanofibers and web to support commercialized PP membranes. They found that the electrolyte uptake was very high, almost 293% in PET/PP (78%/22%) compared to fresh PP separator, which was found to have only 134% uptake. Also, the PP base layer integrated with the porous PET surface melts at high temperatures during shutdown and enhances the performance of the battery as shown in Fig. 13e. This highly ionic conductive and thermally stable PET/PP separator having superior energy storage capability and excellent safety performance could be a promising alternative to the existing commercial LiB separator.

Yang et al. [231] prepared a composite nanofiber separator via electrospinning using low melt PE with high melt polyimide (PI). A multilayered Al_2_O_3_@polyimide/ polyethylene/Al_2_O_3_@polyimide composite membrane was developed where Al_2_O_3_@polyimide layer in outer shells for thermal run-away separator performance and the core with PE layer for low-temperature thermal shutdown property. The Al_2_O_3_@polyimid membrane was prepared by doping Al_2_O_3_ nanoparticles in PI solution and observed excellent conductivity within the multi-layered separator. Additionally, the low melting point of PE has provided very good shutdown properties as tested at 123 °C [231]. The SEM view of the PE membrane before and after heat treatment is shown in Fig. 13b, c. Liu et al. [233] prepared a tri-layered nanofiber composite membrane composed of two sheath layers of SiO_2_ nanoparticles doped polyimide membranes and a core layer of ethyl cellulose modified PE membrane, as illustrated in Fig. 13d. They reported an excellent combination of high thermal stability for SiO_2_ sheath, outstanding mechanical strength, and a low-temperature shutdown function for the PE core of that composite separator [233], and suggested the potentiality of utilizing the developed tri-layered membranes in LiB battery separator for modern electric vehicles.

## Current challenges and potential solutions

The environmental impact of widely used polyolefin materials, has become a major concern due to their contribution to global pollution. More than 400 million metric tons (Mt) of plastic are made around the world every year, and 350 Mt of that plastic ends up as waste. Plastic waste is mostly made up of polyolefin, which makes up about half of all plastic garbage [255]. The non-biodegradable nature of polyolefins presents significant challenges, as materials made from these polymers are considered waste after a few usage cycles. The pollution related to micro- to nano-forms of polyolefin materials, including polyolefin fibers, has become a significant environmental concern. Recent research has highlighted the severe pollution caused by the disposal of polyolefin-based materials, especially in the form of microplastics [256]. Recent studies have shown the harms caused by polyolefin microplastics (PE) in human food. For instance, researchers assume a single person with an exposure of 7.4–50.7 g of microplastics from a PE household chopping board and 49.5 g of microplastics from a PP chopping board [257]. In a different study Hernandez et al. has shown that the progressive development of new plastic packaging to replace traditional paper usage has also create impact on human health by releasing micro and nano plastics during a typical steeping process [258].

Over the past few decades, a variety of studies have been conducted on this subject to address the difficulties related to polyolefin waste materials. The two primary methods for dealing with plastic trash that have arisen are recycling and biodegradation. To establish a circular economy and guarantee a sustainable future, it is imperative to develop effective and sustainable recycling and degrading techniques [259]. Pyrolysis of polyolefins refers to the thermal degradation of these plastics, resulting in the production of several useful compounds. Polyolefin pyrolysis produces a wide range of products through random degradation, with the primary devolatilization reaction producing wax-like compounds with a carbon number range of C20–C50 [260]. This process can lead to the recovery of monomers and the production of liquid hydrocarbons or syngas, which can be further used to prepare olefins, contributing to the circular economy by returning plastic wastes back into the cycle loop [261]. Pyrolysis of plastic waste is considered an alternative way of plastic recovery and a potential solution for the increasing stream of solid waste [262]. Russell and his colleagues produced light olefins and automobile fuel hydrocarbons from waste HDPE using microwaved assisted pyrolysis, offering as an excellent example of chemical recycling [263]. Recycled PE fishing nets are far more crucial for the circular economy for the polyolefin fibers. However, they often have poor properties due to the presence of contaminants and excessive degradation after their lifetime. It is found that incorporating various grades of virgin PE with recycled fishing nets represents a viable method for incorporating these waste products into new one [264].

Microorganisms are the primary component in the biodegradation process that interact with the plastic in various of ways [265]. It was reported by that the thermophilic microbial consortia from cow dung that degrade both low-density polyethylene (LDPE) and HDPE. The degradation of LDPE and HDPE was found to be 75% and 60% correspondingly after 120 days at a temperature of 55 °C. The results of the study demonstrated that LDPE degrades more readily than HDPE, which may be because of the former's higher amorphous content and easier accessibility for deterioration [266]. Awasthi et al. employed the Klebsiella pneumoniae CH001 bacterial strain as a potential enzyme for HDPE degradation. Following a 60-day period, the researchers observed a strength loss of 60% [267]. Xu et al. recently testified on the photo-oxidation degradation of LDPE films with pro-oxidant additive titanium oxide (TiO2) modified by polyethylene glycol (PEG). LDPE, LDPE/TiO2, and LDPE/PEG/TiO2 were used to test the degradation potential. Fungal mycelia and spores were found to grow and colonize profusely on LDPE/PEG/TiO2, but no colonization was seen on the other two films. PEG and TiO2 combine to form a hydrophilic modification that facilitates photooxidation of LDPE, leading in a small molecular weight reduction [268].

One alternative method for dealing with the non-biodegradability of polyolefins is to incorporate biodegradable additives and create functional materials. The development of polymer composites with constant performance characteristics for a certain period, followed by their degradation under the influence of environmental factors, has attached attention. This approach aims to reduce the decomposition period of recycled waste and minimize the territories required for plastic waste [269]. Furthermore, the utilization of natural fiber-reinforced polyolefins has been suggested as a method to reduce the environmental impact of oil-based plastic products. The usage of plant-based fibers in polyolefins not only reduces the number of plastics as the matrix but also reduces the weight of structures, thereby minimizing environmental impact [270]. Moreover, the utilization of natural fibers as reinforcement materials in thermoplastic matrices, such as polyolefins, is driven by growing market trends in terms of environmental impact. The incorporation of various types of fillers into polymer matrices presents an interesting route for producing polymer composites with different properties. However, the environmental impact of these composites and their end-of-life management are critical considerations in addressing the challenges associated with polyolefin materials [271].

In order to address these issues, one potential solution that has been suggested is the implementation of efficient chemical recycling, specifically through the process of pyrolysis, along with the advancement of functionalized polyolefins. Functionalized polyolefins can widen the application window of polyolefins by combining their excellent mechanical properties with the ability to adhere to other materials or create self-assembled or nanostructured materials. This approach holds promise for addressing the pollution related to polyolefin materials and promoting sustainable waste management practices.

## Future scope

Polyolefins are commonly perceived as commodity polymers, have a diverse range of unique features, are inexpensive, and possess a large variety of applications [3]. The chemical nature of polyolefins is more advantageous to other plastics, such as polyurethanes, polyamides, and poly (vinyl chloride) for its very simple composition; comprises only carbon and hydrogen [4]. Polyolefins derive their excellent physical properties from a large variety of molecular chain arrangement with branching. Branching caused by only radical transfer highly influences the physical properties that ensure a variety of olefinic polymer sets with varied useful functionalities, like PP, LDPE, LLDPE, HDPE, UHMWPE etc.[5]. It's useful in a variety of contexts and can be fabricated into many different kinds of potential fibers, including ultrafine nanofiber [2]. Polyolefin fibers have gained recognition as a cost-effective substitute for both natural and synthetic fibers due to their advantageous combination of favorable mechanical characteristics, minimal moisture absorption, and relatively simple manufacturing processes. The gel-spun UHMWPE fibers possess characteristics such as low weight, negligible moisture absorption, and exceptional strength. These attributes place them as formidable contenders against carbon and Kevlar fibers in the realm of protective apparel and high-performance sails [36]. UHMWPE can be utilized as an alternative to carbon fiber in the production of lightweight, high-strength composites for aerospace applications. Furthermore, the UHMWPE gel could be electrospun at high temperatures to create a promising opportunity to produce a distinctive nanofiber with exceptional strength, hence enabling its application in a wide range of diverse fields.

Polyolefins have also evidenced their potentiality as a nanofiber and emerged in a wide area of high-performance applications including filtration and separation, bio-medical, protective clothing and battery separators [129, 132, 169]. The filtration and separation industries make more use of polyolefin nanofibers with diameters as small as possible. In order to make a high-performance nanofibrous filter for water, oil, and air, it is essential to take every measure feasible to reduce the nanofiber diameter. The electrospun PP webs have a high degree of hydrophobicity and exhibit enhanced antibacterial properties. The utilization of appropriate antibacterial nanoparticles during the process of electrospinning has the potential to enhance both the performance and applicability of the resulting materials. In recent years, there has been a significant increase in the utilization of polyolefin-based electrospun membranes or nonwovens as battery separators and shutdown layers in high-demand lithium-ion batteries (LiBs). This trend can be attributed to the favorable mechanical properties and chemical stability exhibited by these materials. Polyolefins are widely recognized as key materials for battery separators due to their exceptional chemical resistance and superior mechanical performance. However, their downsides include poor electrolyte wettability and susceptibility to melting at extreme temperatures, which can lead to internal short-circuits. To address the issue, the researchers fabricated a composite separator consisting of multiple layers of electrospun membranes composed of PET and polyolefin. This composite separator demonstrates enhanced affinity for electrolytes and improved mechanical stability. In addition to PET, various polyolefin-based polymers can be utilized as battery separators to enhance the ultimate performance. This composite separator, which exhibits strong ionic conductivity, thermal stability, improved energy storage capacity, and great safety performance, shows potential as a viable substitute for the currently available commercial LiB separators.

## Conclusions

Derived from the abundant resources of crude oil and natural gas, polyolefins are universally accepted commodity polymers with unique chemical structures. These polymers are commonly recognized as promising high-performance materials in the form of fiber or filament, nanofiber and yarn for numerous applications due to their lightweight, excellent chemical resistance, and amazing physical properties. Polyolefins are demonstrated significant advancements as fiber in micro to nanoscale and emerged as a fascinating material to researchers throughout the globe. The exceptional fiber derived from gel-spun UHMWPE has demonstrated its efficacy in various applications, including medical implants, body armor, and heavy-duty industrial use. Electrospun polyolefin nanofibers also established their potential as an interesting and ingenious material in the field of biomedical engineering for the current and impending applications in heart components, liver parts, dentures to hip, knee joints, tracheal and facial prostheses. The prospective state-of-the-art utilization of polyolefin nanofibers even includes the fabrication of smart textiles, improvement in the performance of lithium-ion batteries, and advancement in wastewater treatment. Accordingly, polyolefins can be utilized to develop new and innovative products that have the potential to revolutionize the way we live and work. However, the challenges of forming functional polyolefin nanofibers using different electrospinning techniques involve a few research studies and reveal an apparent lack of systematic investigation in current scientific literature. Hence, a detailed understanding of the difficulties, including the techniques and relevant factors associated with the development of advanced functional polyolefin nanofibers and comprehending the current state of knowledge to overcome the challenges in forming the nanofibers for the innovative applications have been delineated in this review.

## Data Availability

All data generated or analyzed during this study are included in this manuscript.

## Abbreviations

aPP: Atactic polypropylene
BSA-FITC: Bovine serum albumin labeled with fluorescein isothiocyanate
CAB: Cellulose acetate butyrate
Cu_2_O: Cuprous oxide
DMF: Dimethylformamide
DTAB: Dodecyltrimethylammonium
EHD: Electrohydrodynamic
EVOH: Ethylene vinyl alcohol
FE-SEM: Field emission scanning electron microscopy
HDPE: High-density polyethylene
iPP: Isotactic polypropylene
LDPE: Low-density polyethylene
LLDPE: Linear low-density polyethylene
LNG: Liquefied natural gas
LiB: Lithium-ion battery
MFI: Melt flow index
MFR: Melt flow rate
M-ESP: Melt-electrospinning
Mw: Molecular weight
PB: Poly-1-butene
PE: Polyethylene
PEG: Polyethylene glycol
PDMS: Polydimethylsiloxane
PET: Polyethylene terephthalate
PI: Polyimide
PLA: Polylactic acid
PP: Polypropylene
PP-MNF: PP micro/nanofiber
PPE: Personal protective equipment
PVB: Polyvinyl butyral
PVC: Poly (vinyl chloride)
o-DCB: *Ortho-*Dichlorobenzene
SAN: Styrene-acrylonitrile
SEM: Scanning electron microscopy
S-ESP: Solution-electrospinning
SO: Sodium oleate
t-BAB: Tetrabutylammonium bromide
U: Uranium
UHMWPE: Ultra-High molecular weight polyethylene
